# Cross-dataset benchmarking of machine learning models for marine and atmospheric environmental prediction

**DOI:** 10.1371/journal.pone.0351325

**Published:** 2026-06-12

**Authors:** Xuehua Zhou, Hanming Zhang, Tiantian Du, Quanbo Yuan, Huijuan Wang

**Affiliations:** 1 School of Computer Science and Engineering, North China Institute of Aerospace Engineering, Langfang, China; 2 College of Intelligence and Computing, Tianjin University, Tianjin, China; NRSC: National Remote Sensing Centre, INDIA

## Abstract

Accurate prediction of marine and atmospheric environmental variables is important for climate adaptation, ecosystem management, and operational decision-making, yet practitioners still lack clear guidance on which machine-learning models are reliable across heterogeneous environmental tasks. We therefore developed a unified, leakage-aware benchmark across nine datasets, of which seven passed quality checks for modeling, spanning chlorophyll-a, wind speed, hydrographic observations, biotoxins, and bathymetry, and compared representative linear, tree-based, and sequence models under a common evaluation framework. Results show strong heterogeneity across tasks and model classes: tree ensembles are robust baselines for tabular problems, LSTM-based recurrent sequence modeling is most useful when temporal structure is central, and predictive skill depends more on target structure and covariate quality than on model complexity alone. Within the observational settings represented in this benchmark—predominantly Chinese coastal/estuarine and regional marine datasets, plus one atmospheric reanalysis wind task and one global cast archive—quality-controlled chlorophyll-a is comparatively predictable, whereas event-driven biotoxins and bathymetry inversion remain difficult under the current predictors. These findings provide practical guidance for researchers and environmental monitoring practitioners working in similar data regimes, but they should not be assumed to transfer automatically to untested regions such as the North Atlantic, the Mediterranean, or tropical open-ocean systems without further validation.

## 1. Introduction

Accurate marine and atmospheric environmental predictions are increasingly vital for climate adaptation, sustainable fisheries, and coastal resource management. Reliable forecasts of variables such as chlorophyll-a (Chl-a), wind speed, harmful algal blooms (HABs), and hydrographic conditions support early-warning systems, ecosystem-based management, and operational ocean services [1]. Intensifying climate change and more frequent extremes further amplify the demand for robust predictive tools that can assimilate heterogeneous observations and provide actionable information at relevant spatial and temporal scales [2,3]. These targets also differ substantially in their governing mechanisms and characteristic time scales, so a priori expectations of predictability vary across variables and datasets.

From a process-based perspective, the variables investigated in this study span distinct physical and biogeochemical controls governing predictability. Wind speed at 10 m is primarily driven by synoptic-scale pressure gradients and boundary-layer mixing, and is therefore closely tied to atmospheric state variables available from reanalyses. Surface Chl-a reflects phytoplankton biomass and responds to light availability, nutrient supply, temperature, stratification, and mixing/advection; it often exhibits persistence over days to weeks, so quality assurance (QA) and smoothing can improve signal quality and predictability. By contrast, biotoxin concentrations are produced during episodic harmful-algal-bloom events and depend on species composition, nutrient ratios, temperature–stratification regimes, and transport processes—many of which are unobserved in typical monitoring records. Covariate coverage is especially limited in our biotoxin dataset (two predictors; Table 1), so low predictability is expected under the current feature set.

**Table 1:** Dataset Characteristics and Quality Overview (datasets marked Fail (QA) were excluded from modeling). Benchmark eligibility was determined using the operational inclusion rule described in Section 2.1.2. Expected difficulty is defined a priori from intrinsic data characteristics and is not derived from model outputs. This label is distinct from the empirical skill regimes shown in Fig 6, which are defined post hoc from achieved best-model R². Spatial coverage (coordinate fields and lat/lon bounds) is provided in S7 Table.

Dataset	Samples	Variables	Type	Target	Time Range	Validated	Expected difficulty
rolling_mean	8,855	69	Time Series	Chlorophyll-a (smoothed)	1992–2021	Pass	Easy
cleaned_data	7,819	69	Cross-sectional	Chlorophyll-a	1992–2021	Pass	Medium
era5_daily	102,982	8	Time Series	Wind speed (10 m)	2024–2025	Pass	Medium
processed_seq	8,039	30	Time Series	Chlorophyll-a (processed)	1970–2005	Pass	Medium
hydrographic	4,653	11	Cross-sectional	Chlorophyll-a	2014–2023	Pass	Medium
biotoxin	5,076	2	Cross-sectional	Biotoxin concentration	2013–2023	Pass	Hard
cast	21,865	25	Cross-sectional	Bottom depth	1949–2016	Pass	Hard
phyto_wide	440	46	Cross-sectional	Phytoplankton abundance	2018–2023	Fail (QA)	N/A
phyto_long	82	1	Cross-sectional	Phytoplankton abundance	2018–2023	Fail (QA)	N/A

Each dataset’s type (time-series or cross-sectional), target variable, time span, and validation status are noted.

Finally, bottom depth (bathymetry) is quasi-static, but inferring it from sparse cast-like observations is an under-constrained inverse mapping whose skill depends on spatial coverage and informative proxies, making transfer across regions challenging. These process considerations motivate why we emphasize dataset-specific predictability, baselines, and feature-importance diagnostics rather than treating performance differences as purely algorithmic. Similar links between dominant drivers, persistence, covariate completeness, and achievable skill have also been reported in other engineered environmental prediction settings, including solar power prediction, photovoltaic system monitoring, and PV-environment microclimate prediction [ 4–6].

Traditionally, environmental prediction in marine and adjacent atmospheric settings has relied on empirical or semi-empirical regressions (e.g., ocean-color chlorophyll algorithms), statistical time-series models (e.g., autoregressive integrated moving average (ARIMA) / seasonal ARIMA (SARIMA)-type forecasting), and process-based numerical models [7–9]. These approaches are grounded in mechanistic understanding, but process-based models often require extensive parameterization, high-quality forcing, and substantial computation, which can limit their practical use in data-sparse regions [10,11]. Empirical regressions and classical time-series models may also struggle to capture complex nonlinear relationships between environmental drivers and biological responses.

However, because our benchmark spans heterogeneous targets and feature spaces, no single traditional method can serve as a uniformly matched comparator across all tasks. ARIMA-type models are meaningful mainly for univariate temporal series, whereas widely used empirical ocean-color chlorophyll algorithms require task-specific radiometric/optical inputs that are not shared by most datasets in Table 1. To provide practical context without forcing apples-to-apples mismatches, we therefore add classical baselines only where such comparisons are methodologically meaningful (S12 Table).

In parallel, advances in deep learning (DL) and data-driven Earth system science have demonstrated the potential of modern artificial intelligence to complement process-based understanding and enhance environmental prediction across a wide range of variables [12–15].

Over the past decade, machine learning (ML) and DL methods have been widely applied to Chl-a retrieval, water-quality assessment and HAB prediction from both in situ and remote-sensing data. Tree-based ensembles, shallow neural networks and related algorithms have been trained in rivers, reservoirs, estuaries and coastal waters, typically outperforming traditional regressions or trophic-state indices when relating environmental predictors to Chl-a or bloom indicators [16–21]. Classic ML methodology for these problems includes non-linear ensemble learners, attention-based architectures and robust significance testing procedures [22–25]. At the same time, global observing systems—including satellite ocean-color products, atmospheric reanalyses such as the ERA5 reanalysis (fifth-generation global atmospheric reanalysis) and large in situ archives like the World Ocean Database—now provide massive multivariate datasets that underpin many marine prediction studies [26,27]. For marine wind applications, sequence-oriented DL architectures such as long short-term memory (LSTM) networks and dual-attention convolutional LSTM variants have been used to estimate wind speed over complex terrain and coastal regions [28,29]. More generally, hybrid convolutional neural network (CNN)–recurrent neural network (RNN) designs that combine convolutional feature extraction with temporal memory are widely adopted in other time-series/signal prediction tasks (e.g., outdoor wireless optical communication), reinforcing the broader relevance of these architectures [30]. In riverine and estuarine systems, multi-model ML frameworks that combine in situ and remotely sensed predictors have been developed for long-term eutrophication analysis and Chl-a prediction [31–33]. More broadly, these environmental applications build on general advances in DL theory and practice, as well as established concepts for forecasting skill assessment in geophysical systems [10,34].

In parallel, many regional- and global-scale studies have proposed ML and DL methods for Chl-a inversion and monitoring from satellite ocean-color products, including spatio-temporal models for coastal evolution, convolutional neural networks for global inversion, backpropagation (BP) neural networks for the Yellow Sea, generalized additive models that link Chl-a to environmental and anthropogenic drivers, Transformer-style architectures such as PatchSBSAFormer for long-term open-ocean prediction, and ML methods that invert phytoplankton pigment vertical profiles from satellite data [35–43]. Comparative experiments for western Lake Erie show that gradient-boosted trees and related ensembles can outperform a wide range of alternatives for short-term Chl-a prediction [44,45]. Collectively, these efforts demonstrate that carefully tuned ML/DL models can achieve high skill for specific systems, variables and forecast horizons.

Despite this rapid growth, the studies reviewed above do not yet provide a reusable benchmark for marine and atmospheric environmental prediction. Most are designed around a single region, one target variable, or one sensor product, and are optimized for that specific setting. Their results are therefore informative within each study, but they cannot be directly reused as a common benchmark because preprocessing, feature definitions, train–test split logic, baseline choices, and evaluation metrics differ substantially from paper to paper. In addition, formal uncertainty quantification and statistical sanity checks are often absent or inconsistently reported. As a result, apparent differences in reported skill cannot be attributed confidently to model choice alone, because they may instead reflect easier targets, richer covariates, or more favorable evaluation designs [46–48].

This leaves a clear methodological gap. What is missing is not another successful ML/DL application to a particular environmental variable, but a shared, leakage-aware testbed spanning multiple marine and atmospheric environmental tasks under one evaluation framework: consistent quality control, split strategies matched to data structure, explicit baselines, uncertainty reporting, and statistical sanity checks. Without such a framework, researchers cannot reliably determine which model families remain competitive across tasks, when temporal models add value beyond tabular baselines, or where predictive ceilings are imposed primarily by data quality and covariate coverage rather than by model complexity.

Process-based physical and coupled physical–biogeochemical models face analogous constraints. Even when governing equations are well understood, forecast skill can be bounded by uncertain forcing and boundary conditions, unresolved sub-mesoscale processes, imperfect parameterizations, and sparse observations for initialization and assimilation. In this sense, both mechanistic and data-driven approaches are ultimately constrained by the intrinsic predictability of the target given the available information. Accordingly, we interpret the benchmarked ML/DL skill as an empirical estimate of predictability under a fixed, realistic predictor set—complementary to (rather than a replacement for) process-based modeling.

In this study, we fill this gap by building a unified cross-dataset benchmark rather than another dataset-specific model comparison. Our framework standardizes quality control, preprocessing, split strategies aligned with the data-generating process, baseline-referenced evaluation, uncertainty quantification, and statistical sanity checks, so that model behaviour can be compared across heterogeneous tasks on a consistent basis. This design enables questions that prior case-study literature cannot answer reliably in aggregate: which model families remain strong across tasks, when temporal models provide real added value, and which low-skill regimes reflect intrinsic data and process limitations rather than weak model tuning alone.

Specifically, we apply the same pipeline to nine heterogeneous environmental datasets—predominantly marine, but including one atmospheric reanalysis wind-speed task (era5_daily)—covering Chl-a concentrations, surface wind speed, biotoxin variability, and hydrographic variables, with sample sizes ranging from hundreds to over one hundred thousand records and including both cross-sectional and time-series prediction tasks. We benchmark eight widely used models—including the MEAN baseline, Ridge, least absolute shrinkage and selection operator (LASSO), support vector regression, Random Forest, XGBoost (XGB), LSTM, and a simplified Transformer encoder—under identical data quality control, split strategies aligned with the data-generating process, standardized preprocessing, unified hyperparameter selection, and paired reporting of absolute skill and baseline-referenced skill (baseline R² and ΔR²). This allows us to compare model behaviour across contrasting environmental settings and to interpret performance differences in the context of dataset properties (e.g., intermittency, covariate completeness, and time-series complexity), thereby providing an empirical perspective on predictability under realistic covariate availability that complements process-based expectations.

Importantly, our novelty is not to propose yet another architecture, but to provide a reusable benchmarking framework for a domain where existing studies remain fragmented across targets, regions, and evaluation practices. By turning otherwise isolated case studies into a common testbed, the benchmark makes cross-task comparison, predictability interpretation, and more transferable model-selection guidance possible.

The main contributions of this work are threefold:

1. **Reusable multi-task testbed:** We assemble, quality-control, and document nine heterogeneous environmental datasets spanning marine and atmospheric targets, and categorize them by target variable, predictor set, temporal structure, and expected difficulty, thereby replacing isolated single-system comparisons with a documented common testbed for cross-task benchmarking.
2. **Comparable, leakage-aware evaluation:** We implement a unified evaluation framework combining time-aware splitting, explicit baselines, uncertainty quantification, and statistical sanity checks, reducing the risk of overly optimistic estimates and making cross-dataset conclusions possible in a way that task-specific studies alone do not.
3. **Predictability diagnostics and pre-experiment decision guidance:** By analyzing performance patterns, uncertainty, and feature-importance profiles across tasks of varying difficulty (Table 1), we map a practical predictability spectrum and distill a simple pre-experiment model-selection guide (Table 5) based on measurable dataset characteristics available before model fitting—data structure, sample size, sample-to-feature ratio, persistence/temporal structure, target intermittency or zero inflation, and the presence of immediate proxies. In doing so, we separate model effects from data/phenomenon effects and help bridge the gap between isolated case studies and practitioner-facing model-selection guidance, with temporal recommendations grounded primarily in recurrent sequence-model evidence (especially LSTM) rather than in all DL architectures equally [49].

In addition, we report an exploratory cross-dataset transfer pilot to probe portability across datasets. Because meaningful transfer requires compatible targets and an aligned feature space, we focus on the two closely related Chl-a tasks (cleaned_data and rolling_mean) that share the same predictors but differ in preprocessing and temporal structure; results are summarized in S8 Table.

The remainder of this paper is organized as follows. Section 2 describes the datasets and the unified, leakage-aware benchmarking protocol, including QA, preprocessing, split strategy, model portfolio, baselines, and statistical testing. Section 3 presents the benchmark experiments and overview visual summaries. Section 4 reports the results and provides detailed cross-dataset analyses of performance, robustness, and interpretability. Section 5 discusses implications, limitations, and opportunities for future work, and Section 6 summarizes the main conclusions.

## 2. Materials and methods

**Fig 1:**
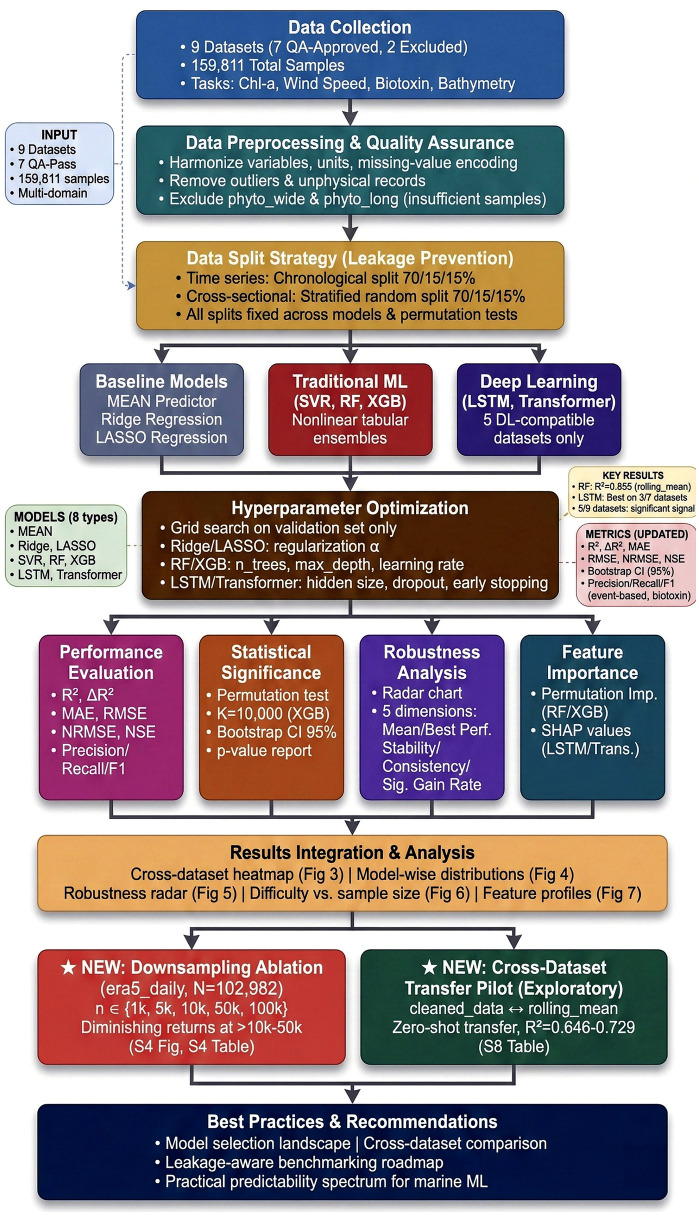
Technical Roadmap.

Our methodology follows a standardized pipeline applied identically to all seven QA-approved datasets (i.e., all except phyto_wide and phyto_long). Fig 1 summarizes the workflow, and S2 Fig provides an expanded benchmarking workflow flowchart from data acquisition to reporting assets: (i) preprocessing and supervised-sample construction, (ii) train–validation–test splitting, (iii) model training and hyperparameter tuning, (iv) evaluation with common metrics and confidence intervals, (v) permutation-based significance testing, and (vi) feature-importance analysis. Applying the same pipeline to all datasets ensures that performance differences are attributable to data and model choices rather than to inconsistent evaluation protocols [25].

### 2.1. Datasets and QA

We assembled nine heterogeneous datasets covering marine and atmospheric environmental prediction tasks. They span Chl-a concentration, surface wind speed, biotoxin concentration and bottom depth, and include both time-series and cross-sectional formats. Together they provide a broad testbed for benchmarking ML and DL models under different signal-to-noise ratios, temporal structures and target types, complementing previous single-system Chl-a retrieval and prediction studies [35,41,45]. Below we briefly summarize each dataset and the applied QA. Most benchmark tasks are marine, but the inclusion of the atmospheric reanalysis wind-speed dataset (era5_daily) means that the study spans both marine and atmospheric environmental prediction settings.

#### 2.1.1. Dataset details.

**rolling_mean**: A time-series dataset (N = 8,855) of smoothed near-surface Chl-a concentrations. The target is a multi-day rolling mean of Chl-a, computed from an underlying daily product. This smoothing reduces high-frequency noise and makes the prediction task comparatively straightforward. Predictors comprise coincident physical and biogeochemical variables (e.g., temperature and salinity), simple calendar descriptors and, for some models, short lags of the target itself. This dataset primarily probes how well models exploit a clean, strongly autocorrelated Chl-a signal.

**cleaned_data**: A cross-sectional dataset (N = 7,819) of quality-controlled, unsmoothed Chl-a observations. Compared with rolling_mean, targets here correspond to single “raw” samples that have passed basic quality-control checks (range checks and removal of obvious outliers), but still exhibit substantial short-term variability. Predictors are similar to rolling_mean. This dataset allows us to directly compare performance on time-averaged versus raw-target prediction and to assess the impact of measurement noise and short-scale variability.

**era5_daily**: A very large time-series dataset (N = 102,982) derived from the ERA5 global atmospheric reanalysis [26]. The target is daily mean 10 m wind speed in a coastal region. In the full benchmark setting, predictors comprise the ERA5 fields latitude, longitude, number, mean sea-level pressure (msl), total cloud cover (tcc), surface solar radiation flux (ssr_flux), and the zonal and meridional 10 m wind components (u10, v10). Because u10 and v10 are immediate physical proxies for scalar wind speed, this task should be interpreted as a proxy-sensitive atmospheric state-estimation problem under realistic reanalysis covariates, rather than as a proxy-free forecast from distal drivers alone. We therefore quantify dependence on these variables explicitly through a predictor-sensitivity ablation (S6 Fig; S11 Table). Because ERA5 already applies internal quality control, we rely primarily on the reanalysis quality-control flags and only remove physically implausible values (for example, negative wind speeds). This dataset tests model scalability to O(10^5) samples with relatively low observational noise.

**processed_seq**: A time-series dataset (N = 8,039) of processed Chl-a sequences constructed from a satellite remote-sensing product. In contrast to rolling_mean, this dataset retains more of the original variability and covers a longer time span, requiring models to capture multi-scale temporal dynamics such as seasonality and interannual changes rather than only short-term autocorrelation.

**hydrographic**: A cross-sectional dataset (N = 4,653) of Chl-a concentration collocated with hydrographic and nutrient measurements from profiling instruments and bottle samples. Predictors comprise temperature, salinity, depth and nutrient concentrations. The high dimensionality and potential multicollinearity can challenge unregularized linear models. In practice, both regularized linear baselines and nonlinear ensembles may be effective, depending on how much of the mapping can be captured by approximately linear relationships under the available predictors.

**biotoxin**: A cross-sectional dataset (N = 5076) of biotoxin concentrations collected from a harmful algal bloom monitoring program. The target distribution is strongly zero-inflated (often zero or near-zero) with occasional large spikes. Predictors comprise a limited set of environmental variables and monitoring metadata. This combination of rare high-concentration events, severe class imbalance in the target distribution, and potential measurement noise suggests that biotoxin prediction is inherently challenging under typical monitoring covariates.

**cast**: A cross-sectional dataset (N = 21,865) of ocean cast profiles aggregated from global hydrographic databases such as the World Ocean Database [29]. The prediction task is to infer bottom depth (bathymetry) from near-surface or profile-averaged hydrographic measurements, which is effectively an inverse problem. Because bottom depth depends on large-scale bathymetric structure and sampling patterns rather than solely on the local hydrographic state, this dataset has weak intrinsic predictability and serves as another “hard” case.

**phyto_wide & phyto_long**: Two phytoplankton community datasets constructed from species- or functional-group abundance records. Under the operational benchmark-inclusion rule defined in **Section 2.1.2**, **phyto_wide** (N = 440, 46 variables; sample/feature ratio = 9.57) did not meet the minimum eligibility threshold for multivariate cross-sectional datasets, and **phyto_long** (N = 82) was too small for stable supervised benchmark evaluation. By contrast, **hydrographic** (N = 4,653, 11 variables) remained eligible. The two phytoplankton datasets are retained in **Table 1** for completeness but were not included in the main benchmark.

#### 2.1.2. QA procedures.

QA was applied uniformly across datasets.

1. Harmonized variable names, units and missing-value encodings, removing records with missing target values.
2. Discarded variables with extensive gaps; imputed sporadic missing values via median imputation (cross-sectional) or short linear interpolation (time series).
3. Removed erroneous or unphysical records and filtered extreme outliers using range checks and visual inspection.
4. Applied a predefined dataset-inclusion rule for benchmark eligibility: after QA, a dataset was retained only if it contained at least 500 observations and, for multivariate cross-sectional datasets, a sample-to-feature ratio of at least 10; datasets with fewer than 100 observations, or failing these criteria, were excluded from the main benchmark (phyto_wide, phyto_long).

Dataset inclusion rule. To ensure that dataset selection is fully reproducible, benchmark eligibility was defined operationally rather than by ad hoc judgment. After QA, a dataset was retained in the main benchmark only if it contained at least 500 observations; for multivariate cross-sectional datasets, we additionally required a sample-to-feature ratio of at least 10. Under our fixed 70/15/15 train–validation–test split, a 500-sample dataset yields approximately 350 training, 75 validation, and 75 test observations, which we treated as the minimum scale for stable model fitting, validation, and held-out evaluation under a common benchmark protocol. Datasets with fewer than 100 observations were considered clearly too small for supervised benchmarking even before considering dimensionality. This rule explains why hydrographic (N = 4,653; 11 variables) was retained, whereas phyto_wide (N = 440; 46 variables; ratio = 9.57) and phyto_long (N = 82) were excluded. Sensitivity analyses supporting this choice are summarized in S1 Fig and S3 Table.

**Fig 2:**
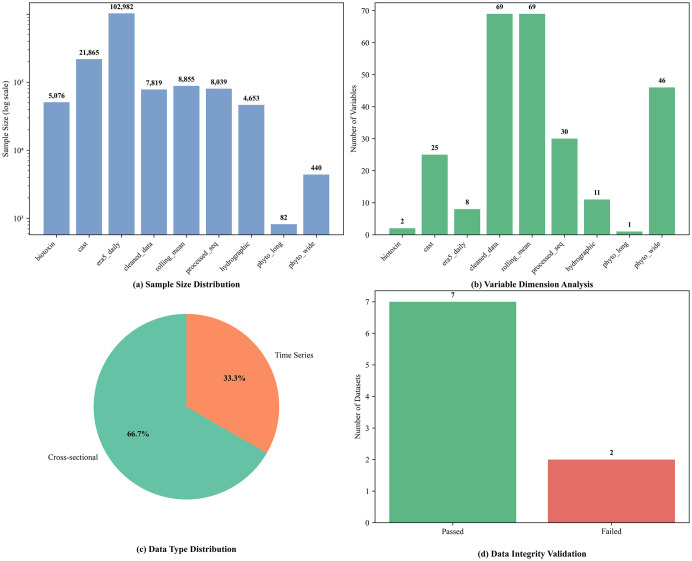
Illustrates key dataset properties. **(a)** Sample size distribution (log scale); **(b)** number of predictors; **(c)** data type (cross-sectional vs time series); **(d)** QA outcomes (datasets passing/failing validation).

Table 1 and Fig 2 summarize the dataset characteristics and QA outcomes. To provide an **a priori** description of task complexity for the benchmark, we assign each dataset an expected difficulty label based only on intrinsic data properties, including: (i) target intermittency / zero inflation and distributional skewness / heavy tails, (ii) temporal smoothness and autocorrelation for time-series targets, (iii) degree of spatiotemporal heterogeneity and sampling inconsistency for cross-sectional data, and (iv) dimensionality relative to sample size and the completeness of physically relevant covariates. Importantly, this expected difficulty label is independent of model outputs. By contrast, Fig 6 later shows empirical skill regimes defined post hoc from achieved best-model R²; it is intended to summarize observed benchmark outcomes rather than to redefine expected difficulty. Achieved predictive skill (e.g., best-model R² and improvements over baselines) is reported separately in the Results (Tables 2–4 and Fig 6) [49].

**Table 2:** Model Performance Comparison and Statistical Significance. To control computational cost, permutation p-values are computed for a single reference model (XGB) as a dataset-level signal sanity check, rather than for every model. Note that ΔR² = R²_best − R²_baseline; because baseline R² can be negative, ΔR² may exceed 1, so it should be interpreted alongside the absolute R² and baseline values.

Dataset	Best Model	Best R²	Baseline R²	Δ vs Baseline	Permutation p (XGB sanity check)	Model Type
rolling_mean	XGB	0.872 ± 0.016	−0.016 ± 0.013	+0.887	p < 1e-4	Traditional ML
cleaned_data	XGB	0.831 ± 0.025	−0.005 ± 0.007	+0.835	p < 1e-4	Traditional ML
era5_daily	RF	0.512 ± 0.030	−0.174 ± 0.021	+0.687	p < 1e-4	Traditional ML
hydrographic	LSTM	0.458 ± 0.083	−0.119 ± 0.049	+0.577	p < 1e-4	Deep Learning
processed_seq	LSTM	0.509 ± 0.056	−0.000 ± 0.002	+0.509	p = 1.0000	Deep Learning
biotoxin	LSTM	0.171 ± 0.052	−0.003 ± 0.007	+0.173	p = 0.9953	Deep Learning
cast	RF	0.383 ± 0.027	−0.000 ± 0.001	+0.383	p < 1e-4	Traditional ML

**Table 3:** Model ranking across datasets.

Rank	Dataset	Best Model	R²	MAE	Type	Improvement¹	Baseline
1	rolling_mean	XGB	0.872	0.012	Traditional ML	+0.887	MEAN
2	cleaned_data	XGB	0.831	0.025	Traditional ML	+0.835	MEAN
3	era5_daily	RF	0.512	0.775	Traditional ML	+0.687	MEAN
4	processed_seq	LSTM	0.509	0.044	Deep Learning	+0.509	MEAN
5	hydrographic	LSTM	0.458	0.146	Deep Learning	+0.577	MEAN
6	cast	RF	0.383	926.629	Traditional ML	+0.383	MEAN
7	biotoxin	LSTM	0.171	14.210	Deep Learning	+0.173	MEAN

¹ Improvement = Best R² – mean baseline R² (absolute difference). Positive values indicate improvement over baseline. All R² values are rounded to three decimals.

**Table 4:** Validation and robustness summary.

Dataset	Samples	Validation	Best R²	Best Model	DL Success Rate^2^	Expected difficulty
rolling_mean	8,855	Pass	0.872	XGB	2/2	Easy
cleaned_data	7,819	Pass	0.831	XGB	2/2	Medium
era5_daily	102,982	Pass	0.512	RF	0/2	Medium
hydrographic	4,653	Pass	0.458	LSTM	2/2	Medium
processed_seq	8,039	Pass	0.509	LSTM	2/2	Medium
biotoxin	5,076	Pass	0.171	LSTM	2/2	Hard
cast	21,865	Pass	0.383	RF	0/2	Hard
phyto_long	82	Fail (QA)	N/A^3^	N/A^3^	0/0	N/A^3^
phyto_wide	440	Fail (QA)	N/A^3^	N/A^3^	0/0	N/A^3^

^2^DL Success Rate = successful runs / total attempts. 2/2: Both LSTM and Transformer successfully applied (produced model results). 0/2: Deep learning models were not applicable or not utilized for this dataset. 0/0: Dataset excluded from analysis (no DL attempted).

^3^N/A: Not applicable – indicates datasets excluded due to QA failure (no performance data), hence no difficulty or model results.

**Fig 3:**
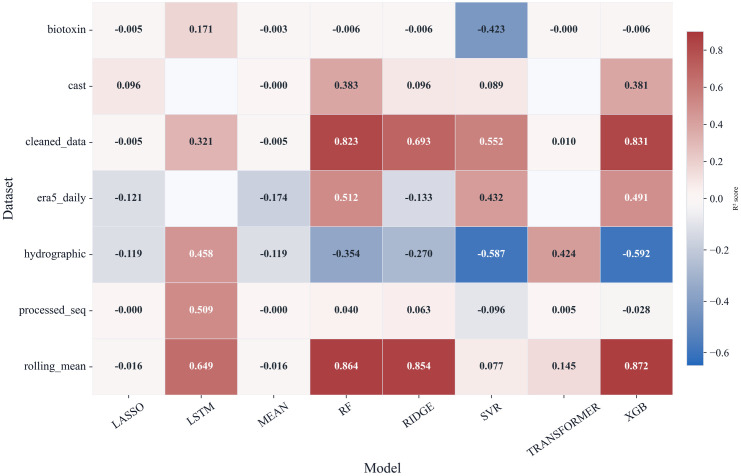
Across-dataset heatmap of test-set R² on seven validated datasets (rows) across seven learning models plus the MEAN baseline (columns). The color scale uses a diverging colormap centered at R² = 0, with the negative range expanded to display the full spread of model failures observed in the benchmark (including severe negative values on hydrographic), so that differences among near-zero, moderately negative, and strongly negative regimes remain visually distinguishable. Blank cells denote model–dataset combinations not evaluated (e.g., deep-learning models were not applied to cast and era5_daily due to data-structure constraints).

**Fig 4:**
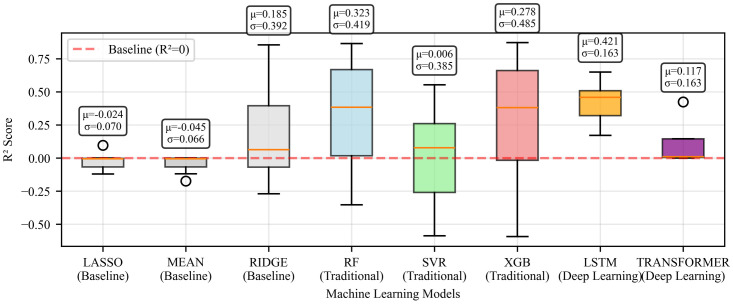
Distribution of test-set R² across datasets for each model (box plots). All metrics are computed on the held-out test set.

**Fig 5:**
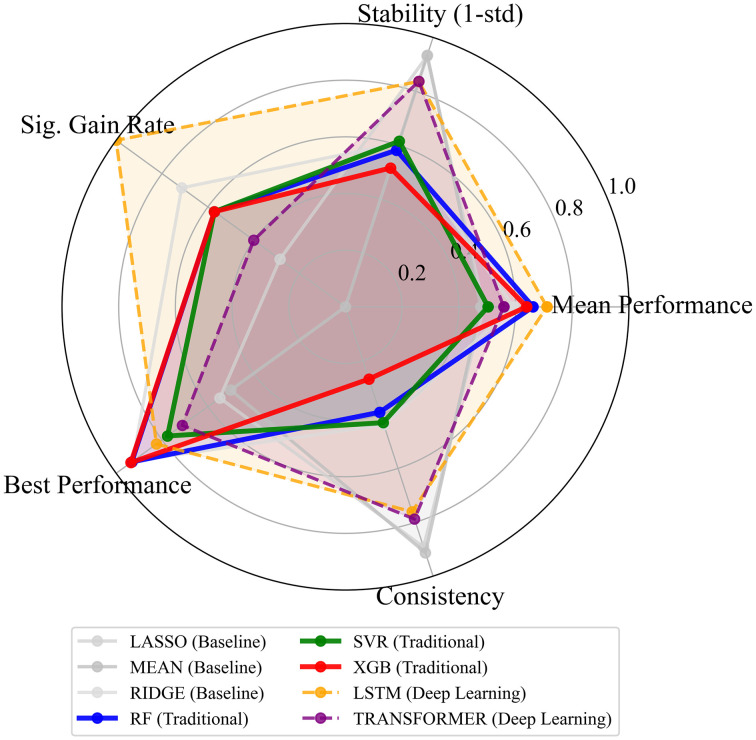
Multi-dimensional robustness summary for the eight models. Sig. Gain Rate is CI-based and does not rely on permutation p-values (see Section 2.4 for bootstrap CI).

The datasets span heterogeneous spatial contexts, ranging from single-location coastal/estuarine monitoring (e.g., biotoxin, hydrographic, phyto_wide) to regional transects/cruise-style observations (e.g., cast) and regional-to-basin-scale gridded products (e.g., era5_daily, cleaned_data, rolling_mean). For each dataset, the presence/absence of latitude–longitude fields, the coordinate variable names, and the latitude/longitude bounds are explicitly documented in S7 Table, enabling spatially informed interpretation of dataset complexity without overloading the main manuscript. This information clarifies whether a dataset originates from a complex coastal/estuarine environment versus a more homogeneous open-ocean/regional setting, which is critical when interpreting predictability differences. Accordingly, the benchmark’s practical recommendations should be interpreted as applying primarily to datasets with similar observational and geographic characteristics to those represented here—namely, predominantly Chinese coastal/estuarine and regional marine monitoring settings, together with one atmospheric reanalysis wind task and one global cast archive—rather than as universal guidance for all ocean basins or climatic regimes.

(a) Sample size distribution (log scale): era5_daily contributes 64% of all samples, while phyto_long has < 0.1%, spanning over three orders of magnitude. (b) Variable dimension analysis: most datasets have 10–70 features, except phyto_long (single variable). (c) Data type: datasets include both cross-sectional and time-series tasks (see Table 1 for dataset-specific labels). (d) QA outcomes: 77.8% “Pass” and 22.2% “Fail (QA)”, highlighting the need to omit small/incomplete datasets for evaluation integrity [25]. Dataset time spans range from multi-decadal (cast, 1949–2016) to near-real-time (ERA5, 2024–2025) (Table 1).

### 2.2. Preprocessing and split strategy

**Preprocessing:** All continuous predictors were standardized to have a zero mean and unit variance with the relevant statistics calculated exclusively on the training set; the same transformations were then applied to the validation and test sets. We inspected target distributions using summary statistics (e.g., skewness) and visual checks (histograms and Q–Q plots) to document potential outliers and extreme skewness, but we did not enforce formal normality tests because most benchmarked models (tree ensembles and neural networks) do not assume Gaussian residuals. Although concentration-type variables are often log-transformed in water-quality modelling to stabilize variance [18,37,39], we did not apply any additional target transformations in our pipeline; instead, we report results in the processed target space provided by each dataset to maintain full reproducibility and avoid introducing dataset-specific offsets. All predictors in the benchmark are continuous, so no categorical encoding was required. Records with missing target values were removed during QA, and remaining sporadic gaps in predictors had already been imputed as described in Section 2.

**Train/validation/test splits and fairness across datasets:** Fairness in this benchmark is enforced within each dataset by applying one split protocol consistently to all models, rather than by imposing the same split type on fundamentally different data structures. For datasets with an explicit temporal order (time series and time-stamped cross-sectional datasets), we applied a chronological split with fixed ratios: 70% of the earliest observations for training, the subsequent 15% for validation, and the most recent 15% for the held-out test set. The exact cutoff timestamps (train_end and val_end) and the corresponding date/index ranges for each dataset are reported in S6 Table to ensure full reproducibility. Using a random split for such datasets would mix earlier and later observations and can yield overly optimistic estimates when temporal dependence, seasonality, or slow covariate drift are present. For purely cross-sectional datasets without reliable timestamps, we used a stratified random split with the same 70/15/15 ratio, where stratification was performed over target-value quantiles to preserve the target distribution across splits. In these cases, a chronological split would be arbitrary and could confound evaluation with incidental record ordering rather than the data-generating process. The split for each dataset was generated once and then kept fixed across all models and all statistical tests (including permutation-based sanity checks), ensuring that model comparisons are fair within each task while the evaluation design remains appropriate to that task type. As an empirical sensitivity check, S9 Table compares chronological and random splits on the time-stamped hydrographic dataset and shows that random splitting yields systematically more optimistic scores (RF: R² −0.354 to 0.548; XGB: −0.592 to 0.861; LSTM: 0.458 to 0.673), supporting our use of chronological splitting as the more conservative, leakage-resistant choice for temporally ordered data.

### 2.3. Machine learning models

We evaluated a portfolio of eight representative ML and DL models that are widely used in environmental prediction [14,34]. Simple reference models serve as baselines: a training-set mean predictor for all datasets, and for time-series tasks an additional persistence model that predicts the last observed value. In Tables 2,3, “Baseline R²” consistently refers to the mean predictor (MEAN) for all datasets to keep ΔR² comparable across tasks. Against these baselines we compare the following models.

1. Mean predictor (MEAN), a simple baseline that predicts the mean target value computed on the training split and applies this constant prediction to the validation/test set. This baseline provides a transparent lower bound on performance under each split and serves as the reference model for reporting relative improvements (e.g., $\Delta R^{\text{2}}$) across datasets.
2. Ridge regression, a linear model with L2 regularization, mitigates multicollinearity by shrinking coefficients toward zero and provides a stable, interpretable baseline for tabular prediction.
3. LASSO regression, a linear model with L1 regularization, encourages sparse solutions by driving some coefficients exactly to zero, which can improve robustness and interpretability when many predictors are weakly informative or redundant. Together, Ridge and LASSO provide strong regularized linear references, but neither can capture the nonlinear interactions or threshold behavior common in marine processes.
4. Support vector regression (SVR) with a radial basis function kernel, which captures smooth nonlinear relationships in moderate-dimensional feature spaces. SVR is capable of modeling nonlinearities but exhibits poor scalability with increasing sample size and is sensitive to kernel and hyperparameter selections for heterogeneous datasets.
5. Random Forests (RF), an ensemble of decision trees trained on bootstrap resamples with random feature subsampling [22]. RF is robust to nonlinearities and interactions, often performs strongly with minimal tuning and is widely used in hydrology and oceanography. As a tabular model, RF does not explicitly represent temporal dependence and can struggle with extrapolation beyond the training domain.
6. Extreme Gradient Boosting (XGB), a gradient-boosted tree ensemble that sequentially fits trees to residuals and has become a de facto standard for tabular ML problems [23]. XGB can model complex nonlinear effects but is more sensitive to hyperparameters and overfitting than RF. Like RF, XGB does not explicitly represent temporal dependence and can struggle with extrapolation beyond the training domain.
7. A long short-term memory (LSTM) network for sequence modeling, which can capture temporal dependencies and has been shown to perform well on environmental time series with strong seasonality and lags [21,34,44]. LSTM can exploit sequential dependence, but its effective memory for very long-range dependencies is limited and performance depends on the quality of the sequence representation.
8. A Transformer-based encoder for sequence modeling, inspired by attention mechanisms originally developed for natural language processing [24,34,45]. This model uses self-attention over patches or windows of the input sequence to learn long-range temporal dependencies. Our simplified Transformer improves scalability for long sequences, but patch/segment tokenization can smooth fine-scale temporal ordering, and training from scratch without pretraining can be disadvantaged on small datasets.

These models were selected to span established strong baselines for tabular geoscience data (tree ensembles and kernel methods) and modern sequence learners (LSTM/Transformer), reflecting commonly used choices in environmental prediction benchmarks [21].

Hyperparameters for all models were tuned on the validation set using coarse grid searches within ranges informed by previous environmental ML studies [18,36,45]. For example, we varied the number of trees and maximum depth for RF and XGB, the regularization strengths for ridge and LASSO, the kernel width and penalty parameter for SVR, the hidden-layer sizes and dropout rates for LSTM, and key architectural parameters for the Transformer encoder (number of layers, attention heads and embedding dimensions). The final hyperparameter settings and all random seeds used in the experiments are documented in the supplementary material.

Practical constraints and scope of the DL baselines: We emphasize that the DL models in this benchmark (LSTM and Transformer) are evaluated as compact, from-scratch baselines under heterogeneous and often data-limited environmental settings. In particular, our Transformer is a simplified encoder-style architecture with patch/segment-based tokenization and reduced depth/width to mitigate overfitting and ensure a fair comparison under limited sample sizes; we do not use large-scale pretraining. Consequently, the Transformer results here should be interpreted as the performance of a lightweight Transformer baseline in our setting rather than an upper bound for the Transformer family.

More generally, DL models typically require a consistent sequence representation and can be data-hungry; on small or weak-signal datasets they may be unstable and susceptible to overfitting. We therefore rely on consistent hyperparameter search, early stopping, and regularization for fairness, and we interpret tree ensembles as the most reliable general-purpose baselines on tabular tasks when sequential structure is weak or absent.

### 2.4. Evaluation metrics

We evaluate predictive skill on the held-out test set using a suite of complementary metrics. For cross-dataset comparability, we use the coefficient of determination (R²) as the primary benchmark indicator and report baseline-referenced improvement (ΔR²). To characterize absolute error, we additionally report Mean Absolute Error (MAE) and Root Mean Squared Error (RMSE), and we compute a Normalized RMSE (NRMSE) by scaling RMSE with the **training-set** target range to avoid test-set leakage. For readers familiar with hydrologic skill scores, we also report the Nash–Sutcliffe efficiency (NSE) as a mathematically equivalent variance-explained measure under the same reference mean. Finally, because some targets are strongly intermittent (notably biotoxin), we complement regression metrics with event-oriented exceedance precision/recall/F1 using a fixed threshold defined as the 90th percentile of the training-set targets. Uncertainty is quantified via 95% bootstrap confidence intervals for test-set R².

**Coefficient of determination (R²).** R² is defined as:


$$\begin{array}{c} R^{\text{2}}=\text{1}-\frac{\sum_{i=\text{1}}^{N}{\left(y_{i}-{\hat{y}}_{i}\right)}^{\text{2}}}{\sum_{i=\text{1}}^{N}{\left(y_{i}-\bar{y}\right)}^{\text{2}}} \end{array}$$ (1)

where $y_{i}$ are observed target values, ${\hat{y}}_{i}$ are model predictions, $\bar{y}$ is the mean of the observed values in the evaluated set, and N is the number of test samples. A value of $R^{\text{2}}=\text{0}$ corresponds to the performance of a mean-predictor baseline (predicting $\bar{y}$), while negative values indicate performance worse than this baseline.

**Complementary error metrics (MAE and RMSE).** Because R² is variance-based and can be less informative for highly skewed or event-driven targets (notably the zero-inflated biotoxin dataset), we additionally report mean absolute error. MAE is defined as:


$$\begin{array}{c} \text{MAE}=\frac{\text{1}}{N}\sum_{i=\text{1}}^{N}|y_{i}-{\hat{y}}_{i}| \end{array}$$ (2)

and RMSE is defined as:


$$\begin{array}{c} \text{RMSE}=\sqrt{\frac{\text{1}}{N}\sum_{i=\text{1}}^{N}{\left(y_{i}-{\hat{y}}_{i}\right)}^{\text{2}}} \end{array}$$ (3)

**Normalized RMSE (NRMSE).** To facilitate comparison across datasets with different scales, we compute NRMSE by normalizing RMSE using the **training-set target range** (to avoid test-set information leakage), as shown in Eq (4):


$$\begin{array}{c} \text{NRMSE}=\frac{\text{RMSE}}{\text{max}\left(y_{\text{train}}\right)-\text{min}\left(y_{\text{train}}\right)} \end{array}$$ (4)

Here, $\text{max}(y_{\text{train}})$ and $\text{min}(y_{\text{train}})$ are computed **only** from the training split (in the same processed target space used for reporting) to prevent information leakage from the test set into metric normalization. The resulting range is fixed per dataset and applied uniformly when computing NRMSE for all models, ensuring fair comparability under a consistent scale reference. If the training-set range is extremely small, NRMSE can become numerically unstable; in such cases we add a small ε to the denominator to avoid division by near-zero values.

**Nash–Sutcliffe efficiency (NSE).** To evaluate the hydrologic skill scores, we calculated the NSE using Eq (5), which is mathematically equivalent to the variance-explained form in Eq (1) when evaluated with the same reference mean:


$$\begin{array}{c} \text{NSE}=\text{1}-\frac{\sum_{i=\text{1}}^{N}{\left(y_{i}-{\hat{y}}_{i}\right)}^{\text{2}}}{\sum_{i=\text{1}}^{N}{\left(y_{i}-\bar{y}\right)}^{\text{2}}} \end{array}$$ (5)

Accordingly, we use “R²” consistently throughout the main text and provide NSE values as a complementary indicator in the Supporting Information.

**Event-oriented exceedance metrics.** We report these for all datasets using a training-defined 90th-percentile threshold, with primary interpretive focus on the event-driven biotoxin task. We additionally report event-based precision, recall, and F1-score. We define an event threshold T as the 90th percentile of the training-set target distribution. An event is observed when y≥T and predicted when $\hat{y}\geq T$. Precision and recall are computed as:


$$\begin{array}{c} \text{Precision}=\frac{\text{TP}}{\text{TP}+\text{FP}} \end{array}$$ (6)

$$\begin{array}{c} \text{Recall}=\frac{\text{TP}}{\text{TP}+\text{FN}} \end{array}$$ (7)

and the F1-score is:


$$\begin{array}{c} \text{F}\text{1}=\text{2}\cdot \frac{\text{Precision}\cdot \text{Recall}}{\text{Precision}+\text{Recall}} \end{array}$$ (8)

where TP, FP, and FN denote true positives, false positives, and false negatives computed on the test set under the fixed threshold T. Importantly, T is determined using only the training split, preventing information leakage.

**Uncertainty and relative improvement.** To assess robustness, we compute 95% confidence intervals (CI) for test-set R² via residual bootstrapping (1,000 resamples), taking the 2.5–97.5 percentile range as the CI. We also report the improvement over a baseline model using:


$$\begin{array}{c} \Delta R^{\text{2}}=\overset{\text{2}}{\underset{\text{model}}{R}}-\overset{\text{2}}{\underset{\text{baseline}}{R}} \end{array}$$ (9)

Where $\overset{\text{2}}{\underset{\text{model}}{R}}$ is the test-set $R^{\text{2}}$ of the evaluated model (or, when reporting the dataset’s peak skill, the best-performing model under our protocol), and $\overset{\text{2}}{\underset{\text{baseline}}{R}}$ is the test-set $R^{\text{2}}$ of the mean-predictor baseline computed using the training-set mean.

Because $\overset{\text{2}}{\underset{\text{baseline}}{R}}$ can be negative on difficult datasets, $\Delta R^{\text{2}}$ may exceed 1; we therefore interpret it jointly with absolute $R^{\text{2}}$ and the baseline value.

**Metric computation space.** All metrics in Eqs (1)–(9) are computed in the processed target space provided by each dataset (no additional inverse transformation is applied). For exceedance-based metrics, the event threshold is computed once per dataset as the 90th percentile of the training-set targets (in the same target space used for reporting), and the same threshold is used for evaluating all models on that dataset to ensure comparability.

### 2.5. Significance testing

We assessed whether observed skill could arise by chance using a label permutation test [23] as a dataset-level sanity check. To control computational cost while using a strong nonlinear reference, we used XGB as a unified reference model across datasets. For each dataset, we performed K = 10,000 permutations while keeping the train/validation/test split fixed. For each permutation, we randomly permuted the target labels across all samples (train+test combined) while keeping the split indices fixed, trained the reference pipeline on the permuted training labels, and evaluated R² on the corresponding permuted test labels. We computed a one-sided Monte Carlo permutation p-value with a + 1 correction:


$$\begin{array}{c} p=\frac{b+\text{1}}{K+\text{1}},b=\sum_{j=\text{1}}^{K}I\left(\overset{\text{2}}{\underset{\text{perm},j}{R}}\geq \overset{\text{2}}{\underset{\text{obs}}{R}}\right) \end{array}$$ (10)

When b = 0, we report p < 1e-4 rather than p = 0. Here $\overset{\text{2}}{\underset{\text{obs}}{R}}$ is the observed test-set R² from the non-permuted data and $\overset{\text{2}}{\underset{\text{perm},j}{R}}$ is the test-set R² from the j-th label permutation. We treat p < 0.05 as evidence that the dataset contains non-chance predictive signal under the XGB reference model. Summary permutation p-values are reported in Table 2 and complete outcomes are provided in S2 Table. These tests help verify that observed predictability is unlikely to arise from chance associations. Because this permutation test is reported at the dataset level using XGB as a unified reference model, it can be conservative for sequence-dominated tasks where the strongest signal is captured primarily by temporal models (e.g., LSTM).

### 2.6. Within-task downsampling ablation (era5_daily)

**Fig 6:**
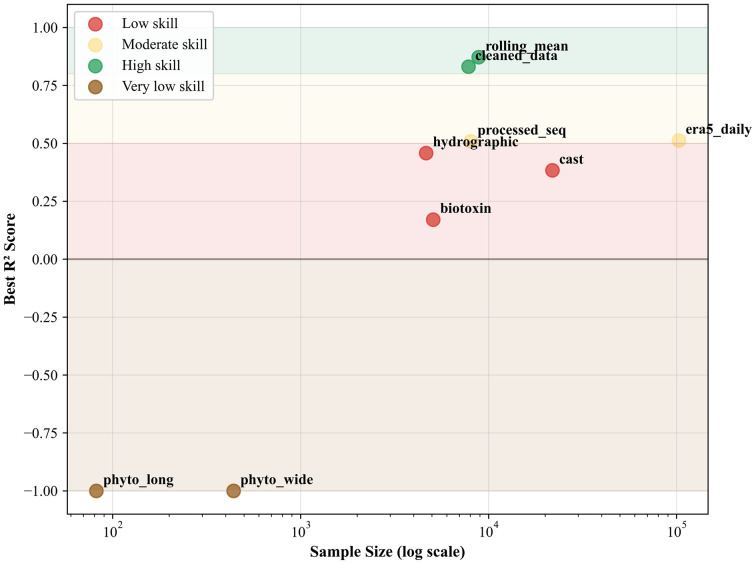
Best test-set R² versus sample size (log scale) across datasets. Colors/background bands indicate empirical skill regimes defined post hoc from achieved best test-set R², rather than the a priori expected-difficulty labels reported in Table 1.

Because Fig 6 compares heterogeneous physical processes and may be confounded, we conducted a controlled ablation on a single task using the largest dataset (era5_daily, N = 102,982). We first randomly subsampled the full era5_daily dataset to size n ∈ {1,000; 5,000; 10,000; 50,000; 100,000} and additionally included the full dataset (N = 102,982). For each subsample, we applied the same chronological split (70%/15%/15%) and evaluated performance on the corresponding held-out test segment. Because subsampling occurs before the split, the test segment is not identical across n; the goal is to measure the size–performance trend under consistent splitting logic. Each subsample size was repeated with multiple random seeds; we report mean test R² and its standard deviation across repeats (S4 Table) and visualize the learning curve (S4 Fig).

### 2.7. Ensuring no data leakage

Given the varied nature of datasets, we took precautions to avoid leakage of information from test to train sets, which can falsely inflate performance [25]. For time-series datasets, as mentioned, strict temporal separation was maintained. For spatial data (like cast profiles), we assumed each sample is independent (random sampling of locations); if spatial clustering was suspected, we would employ a spatial cross-validation, but the metadata did not indicate strong spatial grouping. Feature engineering was minimal and confined to training data only. We also performed a secondary check by shuffling input features; no model achieved high R² under shuffled inputs, providing an additional safeguard against inadvertent leakage. These procedures align with recommended best practices for reliable ML evaluation in environmental sciences.

### 2.8. Experimental setup

**Fig 7:**
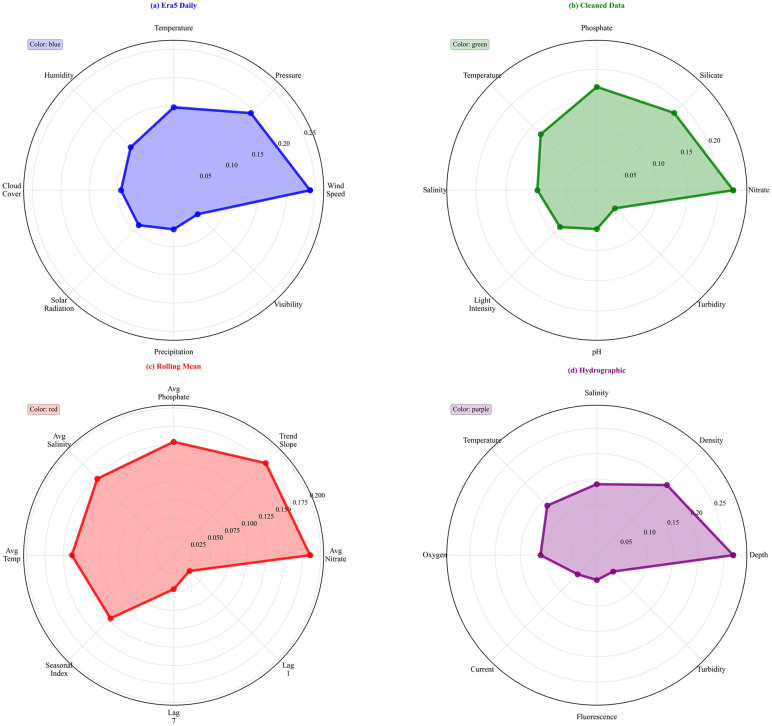
Feature-importance profiles for the best-performing models on selected datasets (e.g., permutation importance or Shapley Additive Explanations (SHAP) values). **All metrics are computed on the held-out test set.** Lag features are computed using past observations only (training/test splits respected) to avoid leakage.

All applicable models were trained and evaluated across the seven validated datasets (excluding the two phyto datasets that failed QA). The experiments were organized in two phases: (1) Performance Benchmarking: training each applicable model–dataset pair to compute R², MAE, and ΔR² vs. baseline (summarized in Table 2 and Figs 3,4); and (2) Robustness & Analysis: further examination of selected models for stability (Fig 5), dataset difficulty relationships (Fig 6), and feature importance (Fig 7). Deep-learning models (LSTM/Transformer) were evaluated on the five datasets for which a sequential input format could be constructed (rolling_mean, cleaned_data, processed_seq, hydrographic, and biotoxin), and were not applied to cast and era5_daily due to data-structure constraints (see Fig 3). All training was conducted on a workstation with Intel Xeon central processing unit (CPU) and NVIDIA RTX 4060 graphics processing unit (GPU); deep models utilized the GPU, while others ran on CPU threads. Training times varied by model complexity and data size – from a few seconds (linear models on small data) to ~30 minutes (Transformer on the largest DL-compatible dataset in our benchmark).

### 2.9. Cross-dataset transfer pilot (exploratory)

To address cross-dataset transfer, we conducted a preliminary “zero-shot” evaluation in which a model trained on one dataset is applied to a different dataset without retraining. Because our benchmark spans different target variables (e.g., wind speed, bathymetry, toxins, Chl-a) and largely non-overlapping predictor sets (Table 1; S7 Table), transfer is only meaningful for dataset pairs that share the same target definition and a compatible feature space. We therefore focused on the two closely related Chl-a datasets, cleaned_data and rolling_mean, which both use the same 69 predictors but differ in target preprocessing (unsmoothed vs smoothed) and data structure/splitting (cross-sectional vs time series).

For each transfer direction (source→target), we trained the model on the source dataset using the same preprocessing and split protocol described above (including fitting standardization on the source training set only), then applied the trained model directly to the target dataset after aligning features by name and applying the source standardization. We report R², MAE, and RMSE on the target evaluation split in S8 Table.

### 2.10. Contextual comparison with classical baselines

To position the benchmark relative to methods already common in environmental practice, we added a limited comparison with classical time-series baselines on tasks where such methods are naturally applicable. Specifically, we evaluated persistence and ARIMA baselines on rolling_mean, processed_seq, and era5_daily, and report the results in S12 Table.

We treat these baselines as contextual rather than as primary benchmark entries for two reasons. First, they are naturally defined only for univariate or quasi-univariate temporal prediction tasks and are therefore not methodologically appropriate for most cross-sectional tasks in Table 1 (e.g., hydrographic, biotoxin, cast). Second, widely used empirical ocean-color chlorophyll algorithms are retrieval models tied to specific radiometric band-ratio inputs and optical assumptions; because most benchmark datasets in this study do not share that input structure, they cannot be used as a single common comparator across the full benchmark.

For rolling_mean and processed_seq, persistence predicts the most recent observed target value, and ARIMA is fit to the univariate target series under the same chronological split used in the main benchmark. For era5_daily, we report a contextual univariate panel-by-date comparison, which is informative for practice but not strictly identical to the multivariate full-feature benchmark used in the main paper. The purpose of S12 Table is therefore not to claim universal superiority of ML over all established oceanographic methods, but to show whether the best benchmarked models provide tangible gains over matched classical baselines where such a comparison is methodologically meaningful.

## 3. Experiment

### 3.1. Across-dataset performance heatmap

To provide an overview of how each model–dataset combination performed, we summarize test-set R² scores in a cross-dataset heatmap (Fig 3). This visualization allows a quick comparison of relative skill across both models and datasets before examining detailed numbers in Table 2.

In the two Chl-a datasets cleaned_data and rolling_mean, tree ensembles achieve the strongest skill (cleaned_data: XGB R² = 0.831, RF R² = 0.823; rolling_mean: XGB R² = 0.872, RF R² = 0.864), while LSTM remains competitive on rolling_mean (R² = 0.649). For era5_daily, the best performance is moderate (RF R² = 0.512; XGB R² = 0.491; SVR R² = 0.432) and linear baselines are negative, consistent with limited feature coverage and nonlinear dynamics. The processed_seq task shows a clear advantage for temporal modeling: LSTM attains R² = 0.509, whereas most tabular models remain near zero. In contrast, hydrographic is challenging for tabular models in this benchmark (e.g., XGB R² = −0.592; SVR R² = −0.587; RF R² = −0.354; RIDGE R² = −0.270; LASSO/MEAN R² = −0.119), while sequence models achieve moderate skill (LSTM R² = 0.458; Transformer R² = 0.424). Accordingly, Fig 3 expands the negative color range so that these severe failures remain visually distinguishable rather than collapsing into a single saturated color band. Finally, biotoxin remains difficult (best LSTM R² = 0.171) and cast achieves moderate skill with tree ensembles (RF R² = 0.383; XGB R² = 0.381).

### 3.2. Model-wise performance distribution

We next examine how each model behaves across all datasets. Fig 4 shows the distribution of test-set R² for each model, highlighting median skill and variability and thereby providing a model-centric view of robustness.

Specifically, each box in Fig 4 represents one model’s distribution of test-set R² across the datasets where it was evaluated (7 datasets for tabular models; 5 datasets for LSTM/Transformer, which were not applied to cast and era5_daily). This highlights model robustness: a model with consistently good performance will have a higher median and tighter interquartile range (IQR). As we will discuss, LSTM shows the highest central tendency on the sequence-capable datasets where it is applied (μ = 0.421, σ = 0.163), while RF and XGB are the strongest among models evaluated on all seven datasets (RF: μ = 0.323, σ = 0.419; XGB: μ = 0.278, σ = 0.485), though performance still varies substantially by task. In contrast, LSTM exhibits comparatively low cross-task spread on the sequence-capable datasets where it is applied, whereas SVR and the linear baselines display the largest variability due to strong dataset dependence. These box plots complement the heatmap by aggregating performance by model rather than by dataset.

### 3.3. Multidimensional robustness profile

To move beyond single metrics, we summarize several robustness indicators for each model in Fig 5. This radar plot combines mean performance, best-case performance, stability, consistency, and significant gain rate into a single multidimensional robustness profile.

Fig 5 summarizes five robustness dimensions for each of the eight models: (i) Mean Performance, defined as the average test-set R² across datasets; (ii) Best Performance, defined as the maximum test-set R² achieved on any dataset; (iii) Stability, measured as 1 − std(R²) across datasets (higher is more stable); (iv) Consistency, defined as the fraction of datasets with a positive gain over the corresponding baseline (ΔR² > 0); and (v) Sig. Gain Rate, defined as the fraction of datasets where the model’s 95% bootstrap CI of R² lies entirely above the baseline’s 95% bootstrap CI (a conservative CI-based improvement criterion). For sequence-dominated tasks, this CI-based criterion can be conservative because the strongest signal may rely on temporal ordering captured more effectively by sequence models. Plotting these on a radar (spider) chart allows comparison of models on a multidimensional robustness profile. For example, LSTM shows the strongest mean performance with a tight spread on the sequence-capable datasets, while RF remains a stable default among models evaluated on all datasets; XGB retains strong peak performance but with higher variability. Such analysis addresses the question of not just how accurate a model is, but how reliable and consistent it is across scenarios [14].

### 3.4. Empirical skill regime versus sample size

To contextualize cross-dataset differences, we relate each dataset’s best achieved test-set R² (Table 2) to its sample size on a log scale (Fig 6). The colored bands in Fig 6 denote empirical skill regimes, not the a priori expected-difficulty labels in Table 1. These empirical regimes are defined post hoc from achieved best-model R² using the thresholds Very low skill (R² < 0), Low skill (0 ≤ R² < 0.5), Moderate skill (0.5 ≤ R² < 0.8), and High skill (R² ≥ 0.8). Their purpose is descriptive: to summarize observed benchmark outcomes on a common scale, rather than to classify datasets in advance. The association between sample size and attainable skill is weak and non-monotonic across tasks: rolling_mean and cleaned_data reach high skill with modest sample sizes (best R² = 0.872 and 0.831), while era5_daily has > 100k records but achieves only moderate skill (best R² = 0.512) and hydrographic remains in the low-skill regime (best R² = 0.458). Conversely, cast attains low-to-moderate skill (best R² = 0.383) despite being a challenging inverse problem, whereas biotoxin remains low-skill (best R² = 0.171). The two phyto datasets (included for reference but excluded from the main benchmark due to QA failure) fall into the very-low-skill regime with strongly negative best R², consistent with insufficient signal and/or extremely limited sample sizes.

Importantly, because Fig 6 compares heterogeneous variables, regions, and predictor sets, it is descriptive rather than causal and should not be interpreted as evidence that “quality outweighs quantity”. To isolate the role of sample size within a single task, we performed a controlled downsampling ablation on era5_daily (S4 Fig; S4 Table). Test‑set skill increases from R² = 0.411 ± 0.038 at 1k samples (subsampled dataset size) to 0.488 ± 0.041 at 5k and 0.524 ± 0.012 at 10k, and then shows diminishing returns (0.542 ± 0.006 at 50k; 0.548 ± 0.001 at 100k; 0.545 ± 0.002 using the full dataset). This confirms that more data improves generalization within a fixed task, while cross-dataset “difficulty” is strongly shaped by intrinsic signal-to-noise and feature informativeness, which set the attainable performance ceiling.

### 3.5. Feature-importance analysis

We next investigate what the models have learned. Fig 7 presents feature-importance profiles for four representative datasets, using the best-performing model on each, so that we can relate predictive skill to physically meaningful drivers.

We employed permutation importance for RF/XGB models [22] and Shapley Additive Explanations (SHAP) values for the LSTM/Transformer model [32] to identify which input features most influenced the predictions. Each subplot in Fig 7 corresponds to one dataset (e.g., hydrographic, rolling_mean, etc.), listing the top features and their relative importance. For example, in the hydrographic dataset, the best-performing sequence model (LSTM) highlights key predictors such as temperature-, salinity-, and depth-related variables, indicating that these hydrographic covariates (together with temporal context captured by sequence modeling) are informative under the benchmark’s chronological evaluation [28]. Feature importance not only aids interpretability but also verifies that models are capturing meaningful relationships. For example, in era5_daily, the RF model identified u10/v10 together with pressure-related variables as key predictors of wind speed; because u10 and v10 are immediate physical proxies for scalar wind speed, their dominance is interpreted together with the dedicated predictor-sensitivity ablation reported in S6 Fig and S11 Table. We discuss the most influential features in the Discussion (Section 5.4).

### 3.6. Reproducibility measures

All experiments were conducted with a focus on reproducibility and fairness. We logged random seeds, library versions, and hyperparameter settings. We found no evidence of data leakage under our preprocessing, split, and sanity-check procedures. The results and Figs described above were generated from the held-out test sets, ensuring an unbiased evaluation. In the next section, we present the results of these experiments in detail, comparing model performance and highlighting key findings with reference to Figs 3–7 and Tables 2–4.

## 4. Results and analysis

### 4.1. Overall model performance

The performance of each model on each dataset is summarized in Table 2 and visualized in Figs 3,4.

Table 2 lists, for each dataset, the best-performing model with test-set R² (±95% CI), the baseline R² (±95% CI), and ΔR². Δ vs Baseline is the improvement in R². The permutation p-value is a dataset-level sanity-check result obtained via a label permutation test using XGB with K = 10,000 permutations on the fixed train/test split (see Methods, Section 2.5 and S2 Table). The smallest resolvable p-value is 1/(K + 1) ≈ 1 × 10^-4; we report p < 1e-4 when b = 0. These p-values indicate whether the dataset contains learnable signal beyond chance and are not model-specific. For processed_seq, p = 1.0000 reflects that this XGB-only sanity check is conservative on a sequence-dominated task and should not be interpreted as the statistical significance of the best model (LSTM). For biotoxin, p = 0.9953 indicates that, under label permutation, the XGB-based dataset-level sanity check cannot distinguish predictive signal from chance on this highly zero-inflated task; this result should not be interpreted as the statistical significance of the best model (LSTM) itself. To clarify what the LSTM actually learns, we additionally report direct prediction diagnostics for the held-out test set (S5 Fig; S10 Table), together with complementary event-oriented metrics (S5 Table). These diagnostics show that the LSTM captures part of the dominant low-to-moderate concentration regime but fails to recover rare high-end events, so the slightly positive R² should be interpreted as limited distribution-level signal rather than practically useful event predictability.

Sensitivity to alternative performance indicators. While Table 2 and Figs 3,4 emphasize R² for cross-dataset comparability, we verified that our qualitative conclusions do not depend on this single metric. S5 Table reports RMSE/NRMSE/NSE for all dataset–model combinations and event-oriented precision/recall/F1 for the biotoxin dataset. Within each dataset, RMSE/NRMSE rankings are consistent with R² because they are derived from the same squared-error residuals, and the main performance patterns remain unchanged: tree-based ensembles remain the most reliable baselines on high-signal tabular datasets, LSTM remains the strongest model on the sequence-dominated task, and the biotoxin task remains difficult under both variance-based and event-oriented criteria (event F1 is near zero under the fixed 90th-percentile training threshold), indicating limited predictability of rare extremes with the current predictors.

### 4.2. Dataset-wise best-model analysis

A closer inspection of S1 Table reveals dataset-specific performance patterns. For the rolling_mean Chl-a series, tree ensembles achieve the strongest skill (XGB R² = 0.872; RF R² = 0.864; RIDGE R² = 0.854), clearly outperforming linear mean/LASSO baselines and highlighting that smoothing plus a rich driver set favors flexible nonlinear models. The cleaned_data dataset, which retains more short-term variability, is slightly harder but still yields high skill with tree ensembles (XGB R² = 0.831; RF R² = 0.823). The modest drop in R² from rolling_mean to cleaned_data indicates the cost of retaining higher-frequency variability, although both tasks remain highly predictable under the available predictors.

On the era5_daily dataset, the best achieved skill is moderate (RF R² = 0.512; XGB R² = 0.491; SVR R² = 0.432), despite the much larger sample size (N = 102,982). This task is best interpreted as a proxy-sensitive atmospheric state-estimation setting, because the full feature set includes u10 and v10, which are immediate physical proxies for scalar wind speed rather than independent distal drivers. To quantify this dependence, we conducted a dedicated predictor-sensitivity ablation using RF and XGB (S6 Fig; S11 Table). In dedicated ablation reruns, removing u10/v10 reduces test-set R² from 0.512 to 0.045 for RF and from 0.491 to 0.098 for XGB, whereas using u10/v10 alone yields intermediate skill (RF: 0.386; XGB: 0.412) that remains below the full-feature setting. These results show that ERA5 performance is not a trivial restatement of the target, but it does depend strongly on immediate wind-component proxies; the remaining non-u/v covariates retain only limited yet non-zero predictive signal. We therefore interpret era5_daily as a useful benchmark task, but not as evidence of proxy-free wind-speed prediction from unrelated meteorological drivers.

The hydrographic dataset is a time-stamped prediction task that remains challenging under the updated benchmark. Hydrographic train–test target distribution diagnostics are provided in S3 Fig to support interpretation of the baseline and model-performance patterns. Only the two evaluated sequence-model baselines achieve positive skill (LSTM R² = 0.458; Transformer R² = 0.424), whereas all evaluated tabular baselines and traditional ML models fall below the MEAN baseline (LASSO/MEAN R² = −0.119; RIDGE R² = −0.270; RF R² = −0.354; XGB R² = −0.592; SVR R² = −0.587). The resulting gain over baseline is substantial for LSTM (ΔR² = 0.577), suggesting that temporal context is essential for extracting signals from this dataset and that instantaneous covariates alone are insufficient.

Split-sensitivity check for time-stamped data. Because the hydrographic dataset is time-stamped and could in principle be partitioned either chronologically or at random, we conducted an explicit sensitivity analysis to examine whether split choice materially affects apparent model performance (S9 Table). The results show that random splitting yields substantially more optimistic test-set scores for all three representative models: RF improves from R² = −0.354 under chronological splitting to 0.548 under random splitting, XGB from −0.592 to 0.861, and LSTM from 0.458 to 0.673. Corresponding MAE and RMSE values also decrease under random splitting. This pattern indicates that random partitioning would give an overly favorable estimate of generalization for this time-stamped dataset, likely by allowing stronger distribution matching between train and test subsets. We therefore retain chronological splitting for hydrographic in the main benchmark as the more conservative and deployment-relevant evaluation protocol.

Similarly, for the processed_seq Chl-a time series, LSTM yields the best performance (R² = 0.509), clearly outperforming non-temporal baselines and most tabular models (RF R² = 0.040; RIDGE R² = 0.063; XGB R² = −0.028). This dataset is intentionally more challenging, retaining higher-frequency variability and potential non-stationarity; the strong relative gain of LSTM supports that sequence models can exploit temporal structure that tree ensembles and linear baselines fail to capture here.

By contrast, the biotoxin dataset is intrinsically difficult: its target distribution is strongly zero-inflated, positive events are sparse and noisy, and only two covariates are available. Even the best model (LSTM) attains only R² = 0.171 and MAE = 14.210. Tabular ML models perform at or below the mean baseline (RF R² = −0.006; XGB R² = −0.006), and SVR fails strongly (R² = −0.423) (S1 Table). These low and often negative R² values indicate that, for most models, predictions do not improve upon a constant mean estimate in variance-explained terms.

To clarify the apparent tension between the slightly positive LSTM R² and the non-significant XGB-based permutation sanity check (p = 0.9953), we examined the LSTM’s held-out test predictions directly (S5 Fig; S10 Table). The diagnostics show that the model does learn part of the dominant low-to-moderate concentration regime: compared with MEAN, RF, and XGB, it reduces MAE (14.210 vs. 17.761–17.778) and RMSE (18.861 vs. 20.718–20.754), yielding a modest positive R². However, the predicted distribution is visibly compressed relative to the observed one, and exceedance diagnostics show no successful recovery of rare high-end events (precision = 0, recall = 0, F1 = 0; TP = 0). In other words, the LSTM appears to learn limited signal in the central mass of the distribution, but not the practically important tail behavior. We therefore interpret the slightly positive R² as evidence of weak distribution-level structure under the current covariates, rather than as evidence of useful event prediction for biotoxins.

Finally, the cast dataset, which seeks to infer bottom depth from hydrographic measurements, remains challenging but is not intractable under the updated benchmark. Tree ensembles achieve moderate skill (RF R² = 0.383; XGB R² = 0.381), while linear models are much weaker (RIDGE R² = 0.096; LASSO R² = 0.096) and the MEAN baseline is near zero. This pattern is consistent with a weak-to-moderate, nonlinear relationship between hydrographic structure and bottom depth: hydrography can encode partial information about depth (e.g., shallow-water temperature/salinity structure), but without explicit spatial coordinates or high-resolution bathymetric covariates, the inverse problem remains underdetermined and sets a clear ceiling on attainable skill.

Contextual comparison with classical baselines. To situate the benchmark relative to methods already common in practice, we compared the best benchmark model against persistence and ARIMA on the three tasks where such classical baselines are naturally applicable (S12 Table). On rolling_mean, persistence attains R² = 0.397 and ARIMA R² = −0.009, both well below the benchmark-best XGB (R² = 0.871). On processed_seq, persistence performs poorly (R² = −0.277) and ARIMA remains near zero (R² ≈ 0.000), whereas the best LSTM reaches R² = 0.509. On era5_daily, ARIMA attains moderate skill (R² = 0.345) and clearly improves over persistence (R² = 0.008), but still underperforms the benchmark-best RF (R² = 0.512). These comparisons indicate that the benchmarked ML/sequence models provide tangible improvement over simple classical time-series baselines on the tasks where a matched comparison is feasible. For era5_daily, this comparison should be interpreted as contextual rather than strictly identical to the main multivariate benchmark, because ARIMA is fit to a univariate panel-by-date formulation rather than to the full covariate set.

### 4.3. Cross-model distribution comparison

Fig 4 provides a complementary, model-centric view by showing the distribution of test-set R² across datasets for each model. Among the models evaluated on all seven datasets, RF attains the highest average cross-dataset performance (μ = 0.323, σ = 0.419; median = 0.383), but with substantial dispersion driven by very high skill on rolling_mean/cleaned_data, moderate performance on era5_daily/cast, and clear failures on hydrographic and biotoxin. XGB shows a lower mean (μ = 0.278) and larger variability (σ = 0.485; median = 0.381), reflecting strong wins on rolling_mean/cleaned_data but negative R² on hydrographic, processed_seq, and biotoxin. LSTM and Transformer are evaluated on five datasets (not applied to cast or era5_daily); within that scope, LSTM is comparatively stable (μ = 0.421, σ = 0.163; median = 0.458) and achieves positive R² on all five tasks, whereas Transformer is lower on average (μ = 0.117, σ = 0.163; median = 0.010) and is often near baseline on several datasets. For biotoxin, model performance is tightly clustered near or below the mean baseline across most methods, consistent with weak cross-sectional signal and the dominance of rare-event unpredictability under the current feature set.

The box plots in Fig 4 also highlight that model ranking depends on which tasks are emphasized and on model coverage. For example, on the two Chl-a datasets (rolling_mean and cleaned_data), XGB/RF dominate, whereas on the explicitly sequence-oriented processed_seq task, LSTM is the clear winner. Hydrographic provides another case where temporal models matter: LSTM/Transformer yield the only positive skill, while tabular models fall below the MEAN baseline. These contrasts highlight the value of cross-dataset benchmarks: conclusions drawn from a single dataset (or from a subset where some model families are not applicable, such as DL on cast/era5_daily) can overstate the general superiority of a given model class [25].

### 4.4. Robustness and stability analysis

Beyond point performance metrics, Fig 5 summarizes robustness along multiple dimensions: Mean Performance, Best Performance, Stability (1 − std), Consistency (1 − (max R² − min R²)/2 across datasets), and Sig. Gain Rate (fraction of datasets where the model’s 95% bootstrap CI of R² lies entirely above the baseline’s 95% bootstrap CI), as well as qualitative training reliability. RF and XGB provide strong overall profiles across the full benchmark but with substantial variability (RF: μ = 0.323, σ = 0.419; XGB: μ = 0.278, σ = 0.485). Relative to the MEAN baseline, RF improves on 5/7 datasets (below baseline on biotoxin and hydrographic), while XGB improves on 4/7 (below baseline on biotoxin, processed_seq, and hydrographic). In contrast, LSTM is evaluated on five datasets (not applied to cast or era5_daily) and shows strong stability within that scope (σ = 0.163) with positive gains over baseline on all evaluated tasks, reflecting a robust profile for sequence-oriented problems.

Transformer models show a more mixed profile: they can be stable on some time-series tasks but generally provide smaller gains and lower robustness indicators than LSTM under the available data volumes. Linear baselines and SVR are computationally reliable but strongly task-dependent: for example, RIDGE performs well on high-signal Chl-a datasets (rolling_mean/cleaned_data) yet fails on hydrographic, and SVR collapses on hydrographic and biotoxin. Overall, Fig 5 supports RF as a reliable default for many tabular environmental prediction problems in this benchmark, while sequence models (especially LSTM) are best deployed when temporal structure is central and model applicability/data support are sufficient [14,15,34].

### 4.5. Predictive skill vs sample size

Fig 6 relates sample size to achieved predictive skill (best-model R²). Its colored bands summarize empirical skill regimes defined post hoc from observed best-model R², whereas Table 1 reports a priori expected difficulty based on intrinsic data properties. Overall, the highest skill is observed for rolling_mean and cleaned_data, whereas era5_daily and processed_seq fall in the moderate-skill regime and hydrographic, cast, and biotoxin remain in the low-skill regime (best R² = 0.458, 0.383, and 0.171, respectively; Fig 6 and Table 3). Importantly, these differences are not explained by sample size alone: some datasets achieve high skill with comparatively fewer samples, while others remain challenging despite large sample sizes. The post hoc skill regimes in Fig 6 are broadly consistent with, but not identical to, the a priori expected-difficulty labels in Table 1; this is expected because Fig 6 summarizes observed benchmark outcomes after modeling, whereas Table 1 summarizes intrinsic task properties before modeling.

These patterns demonstrate that predictability is not a simple monotonic function of sample size. Despite having fewer samples than era5_daily, rolling_mean and cleaned_data are much easier, because the underlying Chl-a signal is strong and the predictors comprise highly informative variables. Conversely, cast has the second-largest sample size but is one of the hardest tasks, due to fundamentally weak relationships between hydrography and bathymetry. Taken together, these results show that sample size is not a sufficient predictor of achieved skill across heterogeneous environmental prediction tasks, because intrinsic signal-to-noise and the availability of key covariates largely determine the achievable ceiling. At the same time, the era5_daily downsampling ablation (S4 Fig; S4 Table) demonstrates that, within a fixed task, increasing training size improves generalization but with diminishing returns beyond ~10k–50k samples. Therefore, “better data” and “more data” should be viewed as complementary levers: quantity helps within-task estimation, while quality/coverage and feature relevance set the ceiling across tasks [14]. We provide the downsampling ablation on ERA5 as a representative large-sample case; extending the same analysis to additional tasks (e.g., chlorophyll retrieval datasets) is a valuable direction for future work.

Tables 3 and 4 summarize the benchmark results and dataset status. Table 3 ranks the seven QA-passed datasets by the best-achieved test-set R² per dataset (i.e., the highest R² attained by any evaluated model on that dataset) and reports the corresponding best-performing model, its MAE, model type, the improvement ΔR² over the baseline, and the identity of that baseline. Table 4 summarizes the dataset validation outcomes and model applicability across datasets: “Pass” denotes datasets that met the QA validation criteria and were included in the benchmark, whereas “Fail (QA)” denotes datasets excluded due to failing QA (e.g., insufficient sample size and/or incomplete coverage). Table 4 also reports the expected difficulty labels defined a priori in Section 2.1.2 based on intrinsic data properties (independent of model performance). Achieved best-model R² values are reported separately as predictive-skill metrics rather than as a definition of difficulty. Accordingly, the empirical skill regimes used in Fig 6 should be interpreted as post hoc summaries of observed benchmark outcomes, not as replacements for the a priori expected-difficulty labels.

Cross-dataset transfer pilot: Beyond the within-dataset benchmark summarized above (Tables 3,4), we performed an exploratory cross-dataset transfer test between the two aligned Chl-a datasets (cleaned_data and rolling_mean; Section 2.9). Zero-shot transfer retains moderate-to-high skill (R² = 0.729 for cleaned_data→rolling_mean; R² = 0.646 for rolling_mean→cleaned_data; S8 Table), but both directions degrade relative to in-domain training on the target dataset (R² = 0.872 on rolling_mean; R² = 0.831 on cleaned_data; Table 3). This indicates a non-trivial domain shift even between closely related datasets (differences in smoothing, temporal structure, and split protocol). For other dataset pairs in our benchmark, direct transfer is unlikely to be meaningful without additional harmonization because targets and/or predictor sets are incompatible (Table 1).

### 4.6. Feature importance & interpretability

Fig 7 summarizes the feature-importance analysis, which serves as an interpretability check and helps relate model behavior to the marine and atmospheric processes represented in the benchmark. For cleaned_data and rolling_mean, top-ranked predictors across RF and LSTM include water temperature, proxies for stratification (such as density or mixed-layer depth), and nutrient-related variables where available. Calendar variables encoding seasonality are also important, consistent with the well-known seasonal cycle of phytoplankton biomass. For hydrographic, depth and temperature emerge as dominant drivers of Chl-a, reflecting light limitation and water-mass structure. For era5_daily, u10/v10 and pressure-related variables dominate the RF importance profile. Because u10 and v10 are immediate physical proxies for scalar wind speed, this ranking should not be interpreted as evidence that the task is free of proxy dependence. Instead, it motivates the dedicated predictor-sensitivity ablation in S6 Fig and S11 Table, which shows that removing u10/v10 sharply reduces skill while still leaving limited non-zero signal in the remaining covariates. For biotoxin, feature-importance patterns are noisier: a small number of environmental indicators receive moderate importance, but no single variable stands out as a clear driver, reinforcing the picture of a weakly predictable, multi-factor system. For cast, surface temperature and salinity show modest importance but cannot fully resolve bathymetric structure, consistent with the low R².

Overall, the feature-importance results are in line with established understanding of marine biogeochemistry and physical oceanography, which increases confidence that the models are capturing meaningful relationships rather than artifacts [2,30]. At the same time, they highlight the limits of interpretability: importance scores reflect associations under the trained model and dataset, and should be complemented by domain expertise and, where possible, mechanistic reasoning.

### 4.7. Key findings summary

In summary, our results can be synthesized into a few key findings:

**Empirical predictability varies sharply across benchmark targets and preprocessing choices.** For biotoxin specifically, direct test-set diagnostics show that the best LSTM model captures limited structure in the dominant low-to-moderate regime but fails to recover rare exceedance events, so its slightly positive R² should not be interpreted as evidence of practically useful event predictability. Within the geographic and observational settings represented in this benchmark, quality-controlled and smoothed surface Chl-a is highly predictable (R² > 0.8 with tree ensembles), whereas event-driven biotoxin variability remains low-skill (best R² = 0.171) and bathymetry inversion remains only moderately predictable (cast best R² = 0.383), consistent with missing drivers, under-constrained mappings, or both at the present resolution. These conclusions should be interpreted as applying primarily to similar coastal/regional monitoring regimes and should not be assumed to hold unchanged in untested basins such as the North Atlantic, the Mediterranean, or tropical open-ocean systems.

**RF is the strongest general-purpose baseline across the benchmark’s tabular tasks.** Across the full benchmark (seven datasets), RF attains the highest average cross-dataset test R² among models evaluated on all tasks (Fig 4) and achieves the best test-set R² on 2 of the 7 datasets (cast and era5_daily), while remaining near-best on several high-skill tabular tasks (e.g., rolling_mean and cleaned_data); however, it underperforms on hydrographic and biotoxin, illustrating that no single tabular baseline dominates all benchmark tasks. This combination of accuracy, stability and modest computational cost, together with RF’s ability to capture nonlinear feature interactions without extensive tuning, supports its use as a default baseline in environmental ML applications across marine and atmospheric settings [14,15]. XGB is similarly competitive overall (Fig 4) and achieves the best test-set R² on 2 of the 7 datasets (rolling_mean and cleaned_data).

**LSTM and recurrent sequence models are most useful when temporal structure dominates.** LSTM outperforms all other evaluated models on 3 of the 7 datasets (processed_seq, hydrographic, and biotoxin), illustrating that recurrent sequence modeling can capture signal that tabular methods miss in explicitly temporal or event-driven settings. In hydrographic, the two evaluated sequence-model baselines deliver the only positive skill (LSTM R² = 0.458; Transformer R² = 0.424), while tabular models fall below the MEAN baseline (S1 Table), suggesting that temporal context is essential under the current predictor set. However, this benefit is not uniform across all DL architectures in our benchmark: the simplified Transformer shows weaker average performance than LSTM across the sequence-capable tasks. Overall, these patterns reinforce that model choice should be matched to task structure and applicability: LSTM-based recurrent models are most valuable when temporal dependencies are central and sufficiently supported by data and evaluation design [14].

**Baselines and statistical tests are essential for trustworthy interpretation.** Baseline R² values can be near zero or negative across datasets (Table 2), underscoring that naïve predictors may fail even when the task appears simple. Using a label-permutation sanity check with K = 10,000 (XGB; Table 2 and S2 Table), we find strong evidence of learnable signal for rolling_mean, cleaned_data, era5_daily, hydrographic, and cast (p < 1e-4). In contrast, biotoxin does not meet p < 0.05 under this conservative XGB-only check (p = 0.9953), and processed_seq also fails the check (p = 1.0000), consistent with sequence-dominated signal that is better captured by temporal models than by tabular XGB.

**Predictability depends on both data quantity and covariate quality.** Where matched classical time-series baselines can be defined, the best benchmarked ML models also provide tangible gains over existing simple practice baselines: XGB/LSTM/RF outperform persistence and ARIMA on rolling_mean, processed_seq, and era5_daily (S12 Table). For era5_daily specifically, predictor-sensitivity analysis shows that performance depends strongly on immediate wind-component proxies (u10/v10), so this task is best interpreted as proxy-sensitive atmospheric state estimation rather than a fully proxy-free forecast from unrelated covariates. Across heterogeneous datasets, sample size alone is not a sufficient proxy for difficulty: moderate-sized but information-rich datasets can achieve high R² (e.g., rolling_mean and cleaned_data), whereas even very large datasets may remain only moderately predictable when key drivers are missing or only partially observed (e.g., era5_daily). A controlled downsampling ablation on era5_daily (S4 Fig; S4 Table) confirms that, within the same task under the same chronological splitting logic, increasing training size improves test-set R² with diminishing returns—so sample size helps approach the achievable ceiling once informative predictors are available.

**Model interpretability broadly aligns with domain science, aiding trust.** Feature-importance patterns highlight plausible drivers for the better-predicted tasks, supporting that models learn meaningful relationships rather than artifacts [32].

**Cross-dataset transfer is only partially feasible under the current data heterogeneity.** A preliminary zero-shot transfer pilot on two aligned Chl-a datasets shows that some skill carries over across datasets, but with clear degradation relative to in-domain training, consistent with domain shift (S8 Table). To make these benchmark-level conclusions usable before full benchmarking, we translate them into a simple pre-experiment model-selection guide based on measurable dataset characteristics (Table 5).

**Table 5:** Practical pre-experiment model-selection guide derived from benchmark evidence. The guide summarizes starting-point recommendations that can be applied before full model comparison. It uses measurable dataset characteristics available at design time, including data structure, sample size, sample-to-feature ratio, temporal dependence, target intermittency or zero inflation, and the presence of immediate proxies. The guide is intended for observational regimes similar to those represented in this benchmark and should be validated locally for unrepresented regions or markedly different data settings.

Situation before modeling	Key measurable characteristics	Recommended starting point	Main caution
Do not benchmark yet	N < 500, or for multivariate cross-sectional data sample/feature ratio < 10	Improve data coverage or reduce dimensionality first	Model ranking likely unstable
Tabular cross-sectional task	No reliable timestamps; adequate N and sample-to-feature ratio	RF first; compare XGB	Keep Ridge/LASSO as linear references
Time-ordered task with clear persistence	Reliable timestamps; strong temporal dependence	LSTM (or another recurrent sequence baseline) + persistence baseline	Use chronological split only
Smoothed / low-noise temporal target	Rolling mean or visibly persistent target	RF/XGB + persistence/ARIMA	Sequence models may be optional
Zero-inflated / rare-event target	Many near-zero values; sparse spikes	Start with strong baselines; use event metrics	R² alone is insufficient
Immediate target proxies present	Predictors include direct components/proxies of target	RF/XGB are practical first choices	Interpret as state estimation, not proxy-free forecasting
Weakly constrained inverse task	Missing key spatial/context variables	RF as a practical benchmark	Feature enrichment may matter more than architecture

**Note:** For time-ordered tasks, chronological splitting should be used whenever the intended application is forward prediction. Stratified random splitting is reserved for purely cross-sectional tasks without reliable timestamps. The guide is intended as a defensible starting point, not a substitute for task-specific validation.

## 5. Discussion

The benchmarking results offer several important insights for the design and deployment of ML and DL models in marine and atmospheric environmental prediction. Below we discuss model performance patterns, the interplay between data quantity and quality, robustness and reliability, interpretability, comparison with prior studies, limitations and future directions.

### 5.1. Model performance patterns

A central result is that tree-based ML and LSTM-based recurrent sequence modeling play distinct roles across the benchmark’s marine and atmospheric prediction tasks. For tabular Chl-a tasks (rolling_mean and cleaned_data), tree ensembles achieve the strongest skill (e.g., XGB is best on both datasets; Table 2), and RF remains highly competitive. On era5_daily and cast, RF provides the best achieved performance among the evaluated models (Table 2), supporting RF as a robust starting point for tabular environmental prediction problems in this benchmark, where nonlinear interactions matter but deep temporal memory is not the primary driver. This is consistent with prior Chl-a prediction and retrieval studies where tree ensembles frequently outperform linear baselines and more complex networks on single-region datasets [36,38,42].

LSTM shows its strengths on tasks where temporal dynamics are central, achieving the best performance on processed_seq, hydrographic, and biotoxin under the models evaluated (Table 2). For hydrographic in particular, the evaluated sequence-model baselines markedly outperform tabular approaches (best R² = 0.458 with LSTM; Table 2), while linear baselines and tree ensembles yield negative R², suggesting that predictive signal is not well captured by cross-sectional covariates alone and that temporal context is critical for this task. These patterns mirror broader findings in environmental ML: recurrent sequence models, especially LSTM-type architectures, can substantially improve skill when the data contain rich temporal structure, but they do not automatically dominate when the key predictive signal is already captured by cross-sectional covariates or when intrinsic predictability is low [14,34,45].

Transformer-based models show a mixed profile in this benchmark. While attention mechanisms can be effective for long-horizon geophysical sequences [34,39,45], in our setting their additional complexity does not translate into systematic gains under the available data volumes and coverage. Overall, the results argue against a “DL by default” approach: tree ensembles (RF/XGB) should be considered strong baselines for tabular environmental prediction tasks in this benchmark, while LSTM-based recurrent architectures are the temporal models most clearly supported by the present results when temporal structure is demonstrably important and the task/model pairing is applicable.

The statements above are still post hoc summaries of benchmark outcomes. To support model choice before full benchmarking, Table 5 translates these findings into a simple pre-experiment guide based on measurable dataset characteristics available at design time: whether the task is cross-sectional or time-ordered, whether reliable timestamps and strong persistence are present, whether the target is zero-inflated or event-driven, whether immediate proxies of the target exist, and whether sample size is adequate relative to dimensionality. The guide is intended as a starting-point heuristic for observational regimes similar to those represented in this benchmark, not as a universal replacement for local validation.

### 5.2. Data quantity vs quality

Data quantity alone does not determine predictive performance. The highest-skill datasets (rolling_mean and cleaned_data) have moderate sample sizes but highly informative predictors and relatively clean targets; as a result, tree ensembles explain more than 80% of the variance (best R² = 0.872 and 0.831, respectively; Table 2). In contrast, cast remains challenging even with a large sample size: while RF/XGB achieve moderate skill (best R² = 0.383; Table 2), the absence of explicit spatial/bathymetric context likely imposes a clear ceiling for this inverse problem.

The medium-skill tasks further illustrate the quantity–coverage trade-off. ERA5_daily provides a very large number of samples, yet the best achieved skill is only moderate (best R² = 0.512; Table 2), consistent with the fact that a limited predictor set only partially constrains daily wind speed. The processed_seq task also falls in the medium regime (best R² = 0.509 with LSTM; Table 2), highlighting that appropriate temporal modeling can be as important as sample count. By comparison, hydrographic shows the opposite pattern: despite adequate sample size, tabular models (including RF/XGB and linear baselines) perform poorly (negative R²), whereas sequence models achieve substantially higher skill (best R² = 0.458 with LSTM; Table 2), indicating that temporal context and feature coverage can dominate over sheer quantity.

This trade-off is clarified by our controlled era5_daily learning-curve ablation (S4 Fig; S4 Table): reducing the subsample size from 100k to 10k and then to 1k decreases mean test-set R² from 0.548 to 0.524 and then to 0.411, while the gains beyond 10k become progressively smaller (full dataset: 0.545; 50k: 0.542; 5k: 0.488). Thus, more data improves generalization within the same task, but it cannot compensate for missing or weakly informative predictors across tasks—highlighting why both quantity and quality/coverage matter.

Our decision to exclude phyto_wide and phyto_long also reflects this principle. Under the operational benchmark-inclusion rule defined in Section 2.1.2, phyto_long fails the minimum sample threshold (N = 82), while phyto_wide fails both the sample-size threshold (N = 440 < 500) and the sample-to-feature criterion (440/46 = 9.57 < 10). These datasets would therefore not support stable supervised benchmarking under the same protocol used for the retained datasets. Incorporating such small datasets would risk drawing spurious conclusions and would violate the methodological rigor we aim for in this benchmark.

### 5.3. Robustness and reliability

Robustness is essential for operational ML in marine science, where models may be deployed under changing conditions and must remain interpretable to non-ML specialists. The radar chart in Fig 5 shows that RF and XGB provide strong overall robustness profiles across the full benchmark, combining high mean/best-case performance with generally consistent gains over the MEAN baseline. However, consistency is not perfect: relative to the MEAN baseline, RF improves on 5/7 datasets (below baseline on biotoxin and hydrographic), while XGB improves on 4/7 (below baseline on biotoxin, processed_seq, and hydrographic). LSTM shows strong stability and positive gains on all evaluated sequence-capable datasets, but it is not applied to cast or era5_daily, so its robustness indicators should be interpreted in light of task coverage. Transformer models are more mixed: they can perform reasonably on some time-series datasets but generally provide smaller gains and can be more sensitive on small datasets. Overall, these patterns support RF as a reliable default for many tabular environmental prediction problems in this benchmark, while sequence models should be deployed when temporal structure is central and the task–model pairing is appropriate.

Permutation-based sanity checks provide an additional guard against overinterpretation. Using K = 10,000 label permutations with XGB (Table 2; S2 Table), we find that rolling_mean, cleaned_data, era5_daily, hydrographic, and cast show clear evidence of predictability beyond chance (p < 1e-4). In contrast, biotoxin does not meet the p < 0.05 threshold (p = 0.9953), and processed_seq also fails this XGB-only sanity check (p = 1.0000), consistent with this check being conservative for sequence-dominated signal that is best captured by temporal models rather than by tabular XGB. These results emphasize the importance of reporting baselines and uncertainty and interpreting low-skill (or low-signal) cases cautiously [25]. The biotoxin LSTM provides a useful example: direct prediction diagnostics (S5 Fig; S10 Table) show that a slightly positive R² can arise from capturing part of the dominant central distribution while still failing completely on rare exceedance events. More generally, benchmark fairness should not be understood as forcing identical split mechanics across all datasets, but as applying the same split protocol to all models within each dataset while matching the split design to the underlying data-generating process.

### 5.4. Interpretability and trust

Interpretability is central to building trust in ML models among domain scientists, managers and stakeholders. The feature-importance patterns observed here (Fig 7) are broadly consistent with existing knowledge of marine biogeochemistry and physical oceanography: temperature, stratification proxies and nutrients matter for Chl-a[48, 48]; wind-related variables matter for wind speed; and depth-related variables matter for hydrographic Chl-a. For era5_daily, however, the dominance of u10/v10 must be interpreted cautiously because these variables are immediate proxies for the target itself. Our new predictor-sensitivity ablation (S6 Fig; S11 Table) confirms that they contribute strongly but do not fully determine performance: removing them leaves low but non-zero skill, whereas using them alone remains weaker than the full-feature model. We therefore treat the ERA5 result as proxy-sensitive atmospheric state estimation under realistic reanalysis covariates, rather than as a proxy-free forecast. This agreement supports the interpretation that the models capture physically meaningful relationships rather than primarily reflecting artifacts or leakage, and it allows domain experts to reason about whether particular predictions make sense [2,33].

At the same time, the noisy and inconsistent importance patterns for biotoxin and cast underscore the limits of interpretability in low-skill regimes. When intrinsic predictability is low and key drivers are missing, importance rankings may fluctuate across models and resamples, and they should not be overinterpreted as causal. Instead, they can be used as exploratory tools to generate hypotheses that must be tested with targeted data collection and mechanistic studies.

### 5.5. Comparison with prior studies

Our findings are broadly consistent with previous ML studies on Chl-a retrieval and prediction, while extending them to a more heterogeneous benchmark setting. For example, earlier works in specific lakes and coastal systems have found that tree-based ensembles or other relatively simple ML models often perform strongly for surface Chl-a retrieval and short-term forecasting [37,42], while DL models provide additional benefits in some long-term or high-resolution sequence settings [34,40,44]. The present benchmark corroborates these patterns but demonstrates them across a more diverse set of tasks, including wind-speed prediction, hydrographic Chl-a relationships, biotoxin occurrence and an inherently hard bathymetric inversion problem.

Furthermore, by applying a unified QA and evaluation pipeline—including time-aware splits, bootstrap confidence intervals and permutation tests—we provide a more stringent assessment than many single-system studies, where differences in pre-processing and validation make cross-study comparisons difficult. Our results thus help to bridge isolated case studies and offer a more generalizable picture of when particular model classes are likely to succeed or struggle in environmental applications spanning marine and atmospheric settings. To further connect the benchmark to established practice, we added contextual classical baselines (persistence and ARIMA) on the three time-series tasks where such comparisons are methodologically meaningful (S12 Table). On all three tasks, the benchmark-best ML or sequence model outperforms these classical baselines, sometimes by a wide margin (e.g., rolling_mean and processed_seq), while on era5_daily the ARIMA baseline remains competitive but still below RF. We emphasize, however, that this does not establish universal superiority of ML over all oceanographic practice: process-based numerical models and empirical ocean-color retrieval algorithms are task-specific and require harmonized forcings or optical inputs not shared across the full benchmark. The comparison is therefore best understood as evidence that the benchmarked ML models provide tangible gains over matched simple classical baselines where such a comparison is feasible. Accordingly, this “more generalizable picture” should be understood relative to the benchmark’s represented observational regimes, not as a universal ranking that automatically extends to all marine basins or climatic zones.

### 5.6. Limitations

Several limitations of the present study should be acknowledged. First, while our benchmark covers diverse, predominantly marine contexts plus one atmospheric reanalysis setting, its spatial footprints remain heterogeneous and not globally uniform; the dataset-specific spatial extents are summarized in S7 Table. In practice, the benchmark is dominated by Chinese coastal/estuarine and regional marine observation settings, supplemented by one global atmospheric reanalysis wind task and one global cast archive inverse problem. Our practical recommendations should therefore be interpreted as applying primarily to similar observational regimes, covariate sets, and hydrographic contexts. We do not, for example, include dedicated benchmark tasks from the North Atlantic, the Mediterranean, or tropical open-ocean systems, nor do we include global high-resolution satellite imagery, detailed nutrient fields, or in situ optical properties that might materially change relative model performance. Second, our model portfolio, while representative, is not exhaustive: convolutional neural networks for gridded data, graph neural networks for irregular observation networks, and more sophisticated physics-guided architectures were not systematically evaluated here [14,15,34]. Additionally, process-based physical or coupled physical–biogeochemical models were not included in the benchmark. Such models encode conservation laws and mechanistic constraints, but their practical skill depends strongly on harmonized forcing and boundary conditions, spin-up/initialization, and (often) data assimilation choices. A fair end-to-end comparison would therefore require aligned inputs and careful treatment of these configuration factors, which is beyond the scope of the present multi-dataset ML benchmark. We instead view the reported ML/DL performance as a data-driven reference for achievable predictability under a common observational feature set.

Third, hyperparameter tuning was performed using coarse grid searches and a single validation split per dataset. More advanced tuning strategies (e.g., Bayesian optimization or nested cross-validation) might further improve some models but would substantially increase computational cost. Fourth, we focus on point predictions and do not model uncertainty beyond bootstrap confidence intervals, nor do we evaluate fully probabilistic forecasts.

Finally, while we took care to avoid temporal leakage and to use held-out test sets, our assessments remain conditional on the specific periods and data sources considered; generalization to new regions or strongly non-stationary future climates is not directly tested. Accordingly, the model-selection guidance reported here should be viewed as benchmark-conditioned rather than universally transferable across all ocean basins. We additionally report a limited “zero-shot” cross-dataset transfer pilot for two aligned Chl-a datasets (cleaned_data ↔ rolling_mean; S8 Table), showing that transfer can be non-trivial even for closely related datasets due to domain shift (e.g., smoothing, temporal structure, and split protocol). More general transfer across mismatched targets/predictors would likely require harmonized covariates and explicit domain-adaptation strategies.

### 5.7. Outlook – toward hybrid and ensemble approaches

Future work can build upon this benchmark in four main directions.

**First (data enrichment and harmonization),** progress is likely to be driven less by marginal architectural changes and more by adding process-relevant covariates and improving spatiotemporal coverage. For Chl-a tasks, high-priority additions include satellite-derived variables (e.g., multi-spectral ocean-color reflectance products, sea surface temperature (SST), photosynthetically active radiation (PAR) / light proxies), mixed-layer or stratification indicators, and hydrodynamic forcings (winds/currents where available). For the biotoxin task, stronger predictability likely requires covariates that reflect HAB drivers and transport (e.g., nutrient loading proxies, salinity/river discharge indicators, bloom intensity proxies from satellite, and basic circulation/residence-time descriptors), and reframing into event-aware learning (e.g., a two-stage model: occurrence classification followed by magnitude regression). For bathymetry inversion, incorporating explicit spatial variables (latitude/longitude, distance-to-coast, regional context) and spatially structured priors is a clear priority, as the mapping is otherwise under-constrained.

**Second (hybrid ML + mechanistic modeling),** physics-guided ML can embed conservation or mass-balance constraints and can also leverage mechanistic model outputs as informative priors—for example, using residual learning (ML predicts corrections to a mechanistic baseline), emulator-style surrogates for expensive process models, or data-assimilation–inspired hybrids that combine observational features with dynamical consistency. This is especially relevant for low-predictability regimes (biotoxin and bathymetry inversion) where key processes are only partially observed.

**Third (architecture matched to data geometry),** beyond RF/LSTM, promising directions include CNN/ConvLSTM-style models for gridded satellite products, graph neural networks for irregular station networks, and probabilistic predictors that output calibrated uncertainty for decision support—each evaluated under the same leakage-aware protocol.

**Fourth (transfer and domain adaptation),** systematic cross-dataset generalization will likely require shared predictor spaces and explicit adaptation strategies. Practical routes include pretraining on large-scale satellite time series and lightweight fine-tuning, as well as domain-shift diagnostics to quantify when portability is feasible versus when new covariates or region-specific calibration are required.

## 6. Conclusions

We benchmarked eight representative ML/DL model families across nine heterogeneous environmental datasets—predominantly marine, but including one atmospheric reanalysis wind-speed task—of which seven passed QA for modeling. The benchmark used a unified, leakage-aware protocol with time-aware train–validation–test splits, bootstrap confidence intervals, and a conservative XGB-based permutation sanity check (K = 10,000).

Achievable skill is governed primarily by data/phenomenon structure, revealing a practical predictability spectrum within the geographic and observational settings represented in this benchmark: QA/smoothing yields high predictability for surface Chl-a under common covariates, whereas rare-event biotoxins remain low-skill (best LSTM R² = 0.171); moreover, the conservative XGB-based dataset-level permutation sanity check does not support strong non-chance signal (p = 0.9953), and direct test-set diagnostics indicate that the model captures only limited structure in the dominant low-to-moderate regime rather than useful rare-event predictability. These conclusions should be applied primarily to similar coastal/regional monitoring regimes and should not be assumed to transfer unchanged to untested basins such as the North Atlantic, the Mediterranean, or tropical open-ocean systems without additional validation.

Model choice should match data geometry: tree ensembles (RF/XGB) are robust, low-tuning baselines for tabular signals, while LSTM-based recurrent models add the clearest value when temporal state evolution dominates (hydrographic: best R² = 0.458 with LSTM versus negative tabular baselines). This benefit should not be generalized to all DL architectures in our benchmark, because the simplified Transformer shows weaker average performance than LSTM across the sequence-capable tasks. To make this guidance actionable before model fitting, Table 5 converts these post hoc benchmark patterns into a practical pre-experiment decision aid based on measurable data characteristics such as data structure, sample size, persistence, zero inflation, and proxy dependence. Where matched classical time-series baselines are available, the best benchmarked models also provide clear practical gains: XGB, LSTM, and RF outperform persistence/ARIMA on rolling_mean, processed_seq, and era5_daily, respectively (S12 Table), although this contextual comparison should not be overgeneralized to all process-based or task-specific empirical oceanographic methods. For era5_daily, predictor-sensitivity ablation shows that the reported skill depends strongly on immediate wind-component proxies (u10/v10), so this result should be interpreted as proxy-sensitive atmospheric state estimation rather than fully proxy-free wind-speed prediction.

Data quantity alone is not sufficient: within-task downsampling on era5_daily shows diminishing returns with increasing training size, and an exploratory transfer pilot between aligned Chl-a datasets indicates non-trivial domain shift.

The most impactful next steps are covariate enrichment (satellite and hydrodynamic/nutrient proxies), explicit spatial/context features for inverse problems, event-aware learning for intermittent targets, and hybrid physics–ML or domain-adaptation approaches for low-predictability regimes.

## Supporting information

S1 Fig Small-sample dataset analysis. Sensitivity of benchmark eligibility to small-sample exclusion thresholds.
(TIF)

S2 Fig Benchmarking workflow flowchart. End-to-end pipeline from data acquisition to reporting assets.
(TIF)

S3 Fig Hydrographic target distribution diagnostics. Train vs test distribution comparison for baseline interpretation.
(TIF)

S4 Fig ERA5_daily downsampling learning curve (full-dataset subsampling before the chronological split). The curve shows mean test-set R² over repeated subsamples; shading indicates ±1 standard deviation (SD).
(TIF)

S5 Fig Held-out biotoxin test-set prediction diagnostics for the LSTM model. Raw-scale and log1p-scale observed-versus-predicted scatter plots, observed/predicted distribution comparison, and exceedance confusion matrix for the best LSTM model on the held-out biotoxin test set. The diagnostics show that the model captures limited structure in the dominant low-to-moderate concentration regime but fails to recover rare high-end exceedance events.
(TIF)

S6 Fig ERA5 proxy-sensitivity ablation. Test-set R² under the full covariate set, without u10/v10, and with u10/v10 only, showing that ERA5 performance depends strongly on immediate wind-component proxies but is not entirely reducible to them.
(TIF)

S1 Table Full model performance matrix. Test-set R² with 95% bootstrap confidence intervals across datasets and models, with dataset-level permutation p-values (XGB; K = 10,000) reported as a sanity check; p-values are computed per dataset and therefore shared across models within the same dataset.
(DOCX)

S2 Table Permutation test results (K = 10,000; XGB). Dataset-level label-permutation sanity-check p-values using XGBoost with K = 10,000 permutations, reflecting overall dataset predictability rather than model-specific significance.
(DOCX)

S3 Table Operational benchmark-inclusion threshold analysis for small or sparse datasets. Summary of sample size, dimensionality, sample-to-feature ratio, and benchmark-eligibility status for datasets near or below the predefined inclusion threshold, showing why phyto_long and phyto_wide were excluded from the main benchmark and retained for descriptive analysis only.
(DOCX)

S4 Table RA5_daily downsampling results using full-dataset subsampling before the chronological split. Reported values are mean test-set R² and standard deviation across repeats for each subsample size.
(DOCX)

S5 Table Alternative evaluation metrics on the held-out test set. RMSE, NRMSE (normalized by the training-set target range), and NSE for all dataset–model combinations; For all datasets, event-based precision/recall/F1 using a fixed threshold defined as the 90th percentile of the training-set target distribution (evaluated on the test set; processed target space).
(DOCX)

S6 Table Dataset split summary. Cutoff dates and train/validation/test ratios for all time-series datasets.
(DOCX)

S7 Table Geographic scope summary. Region descriptions and coordinate bounds for each dataset. For datasets without geolocation metadata (e.g., site-aggregated or non-georeferenced products), coordinate fields are reported as NA in S7 Table.
(DOCX)

S8 Table Cross-dataset transfer pilot results. Performance of a model trained on one chlorophyll-a dataset and evaluated directly on another (cleaned_data ↔ rolling_mean), reporting R²/MAE/RMSE and illustrating the impact of domain shift on predictive accuracy.
(DOCX)

S9 Table Split-sensitivity analysis for the time-stamped hydrographic dataset. Test-set performance of RF, XGB, and LSTM under chronological versus random 70/15/15 splits, showing that random splitting yields systematically more optimistic scores.
(DOCX)

S10 Table Biotoxin test-set diagnostic summary across baseline and representative models. Test-set sample size, threshold, R², MAE, RMSE, and exceedance precision/recall/F1 for MEAN, RF, XGB, and LSTM on the biotoxin dataset. For the LSTM, the test-set count is slightly smaller because sequence construction removes a small number of boundary samples near the split edges.
(DOCX)

S11 Table ERA5 proxy-sensitivity ablation results. Test-set R², MAE, RMSE, and ΔR² relative to the full-feature setting for RF and XGB under three ERA5 predictor settings: full covariates, without u10/v10, and u10/v10 only.
(DOCX)

S12 Table Contextual comparison with classical time-series baselines. Test-set performance of persistence and ARIMA on rolling_mean, processed_seq, and era5_daily, together with the corresponding best benchmark model for reference. For era5_daily, the comparison is contextual because ARIMA is fit to a univariate panel-by-date series rather than to the full multivariate covariate set.
(DOCX)
