# Supplementary figures and images for: Cross-dataset benchmarking of machine learning models for marine and atmospheric environmental prediction
Source: PLoS One. 2026 Jun 12;21(6):e0351325. doi: 10.1371/journal.pone.0351325 (PMC13262816; doi:10.1371/journal.pone.0351325)

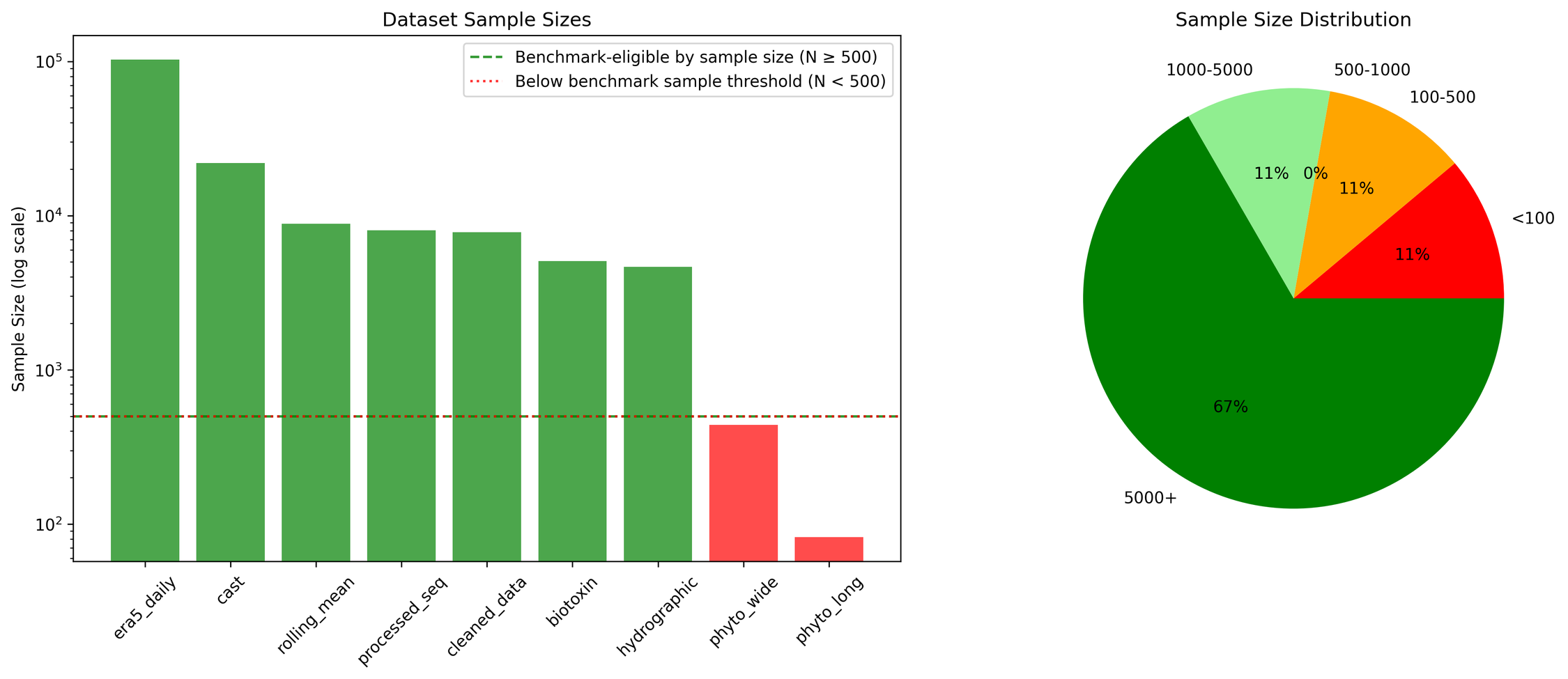

Supplement: S1 Fig — Sensitivity of benchmark eligibility to small-sample exclusion thresholds. (TIF) [file pone.0351325.s001.tif]

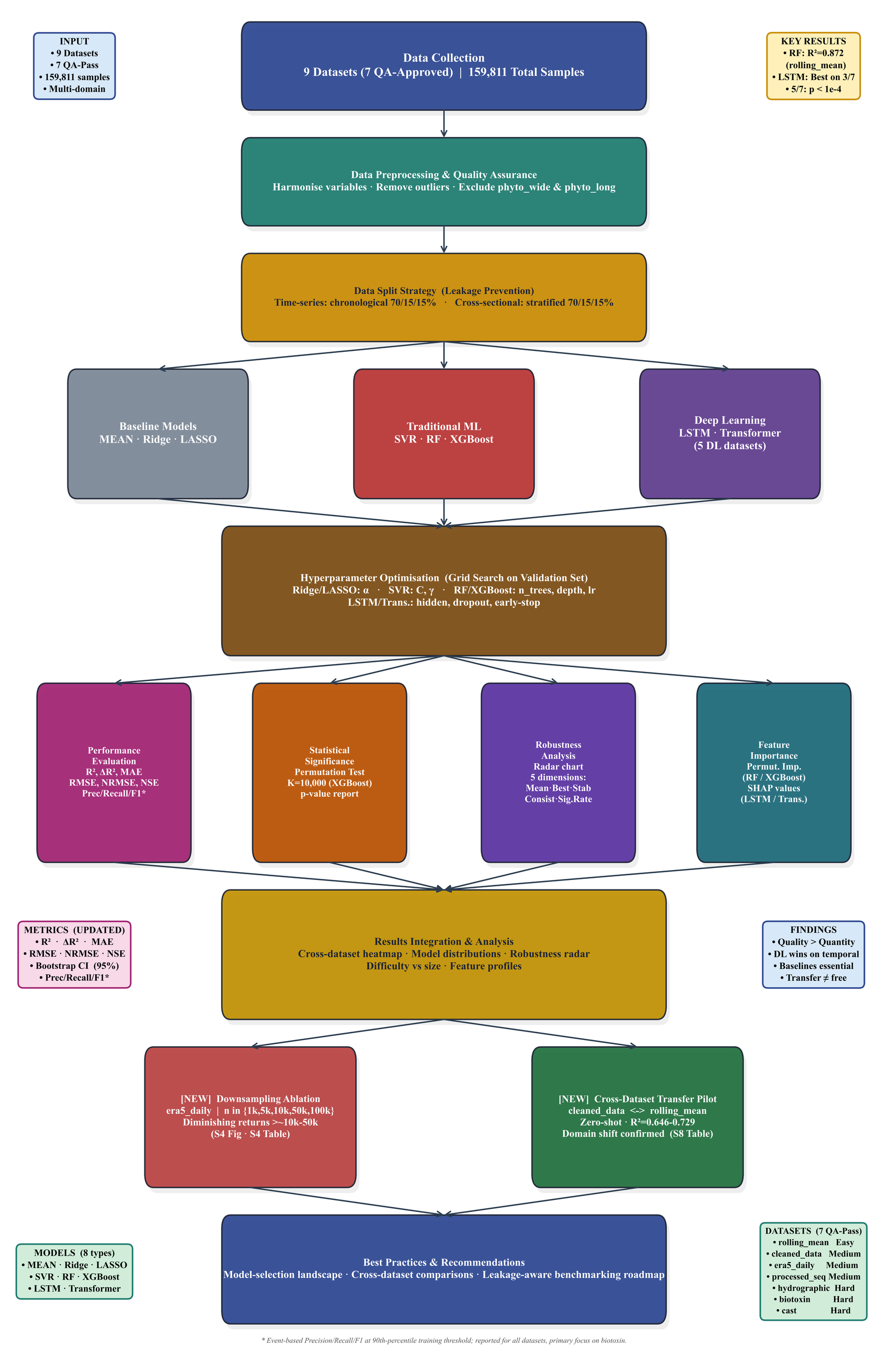

Supplement: S2 Fig — End-to-end pipeline from data acquisition to reporting assets. (TIF) [file pone.0351325.s002.tif]

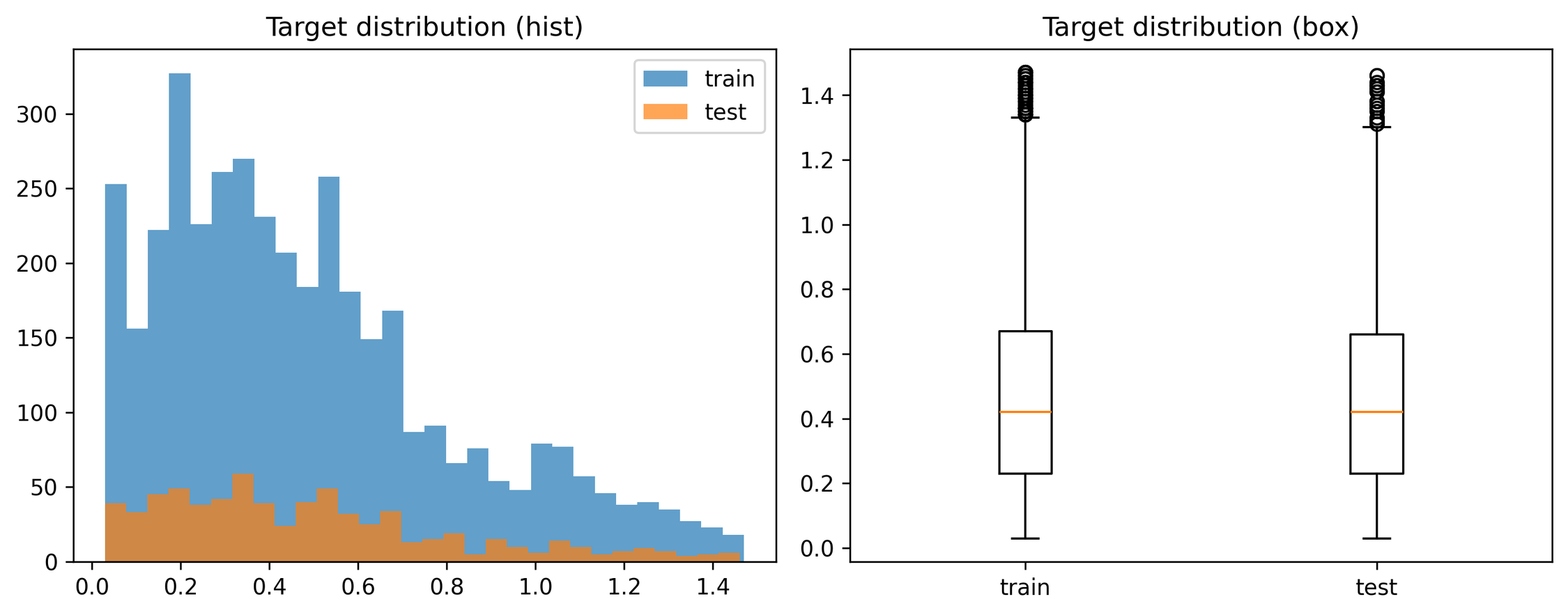

Supplement: S3 Fig — Train vs test distribution comparison for baseline interpretation. (TIF) [file pone.0351325.s003.tif]

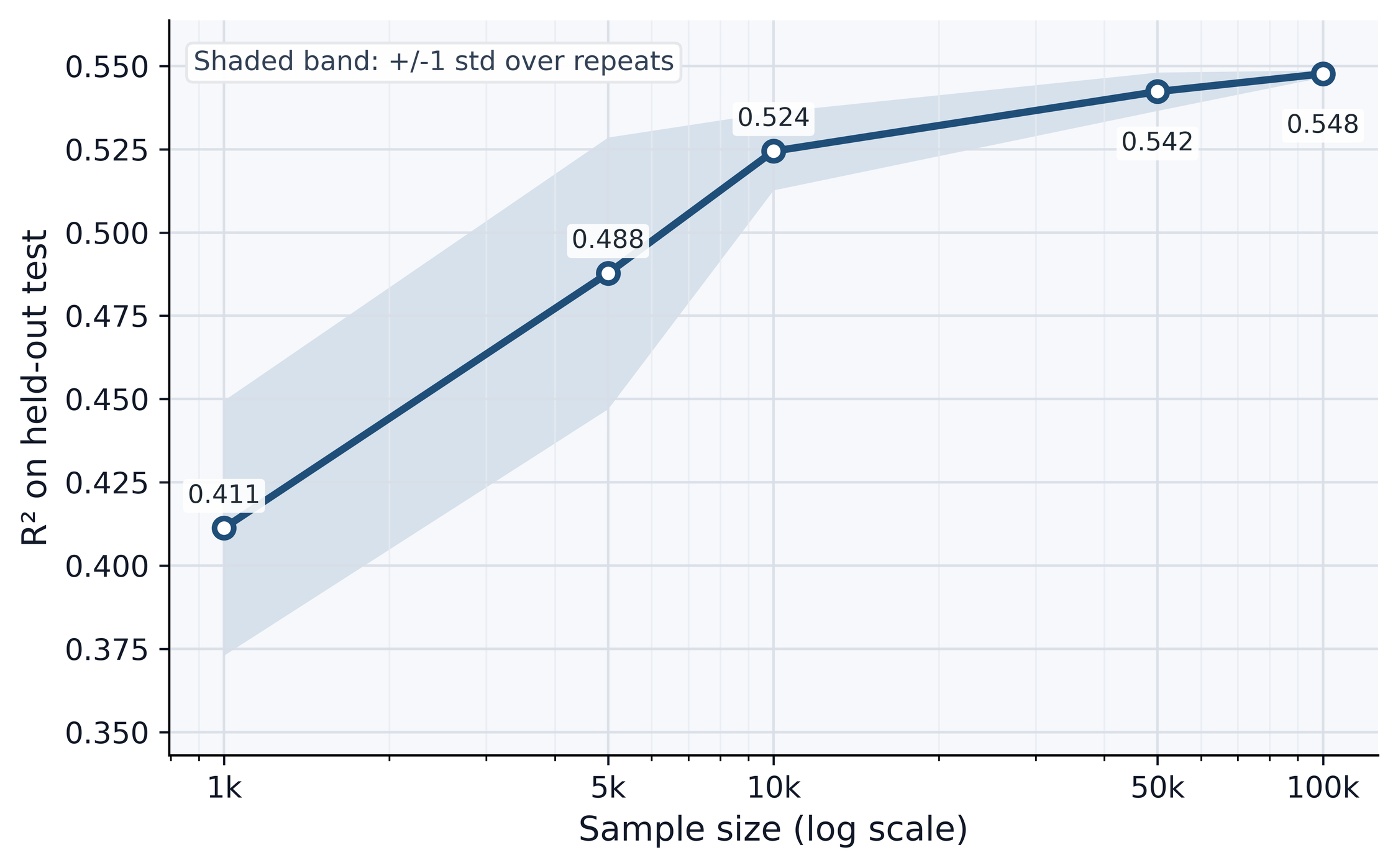

Supplement: S4 Fig — The curve shows mean test-set R² over repeated subsamples; shading indicates ±1 standard deviation (SD). (TIF) [file pone.0351325.s004.tif]

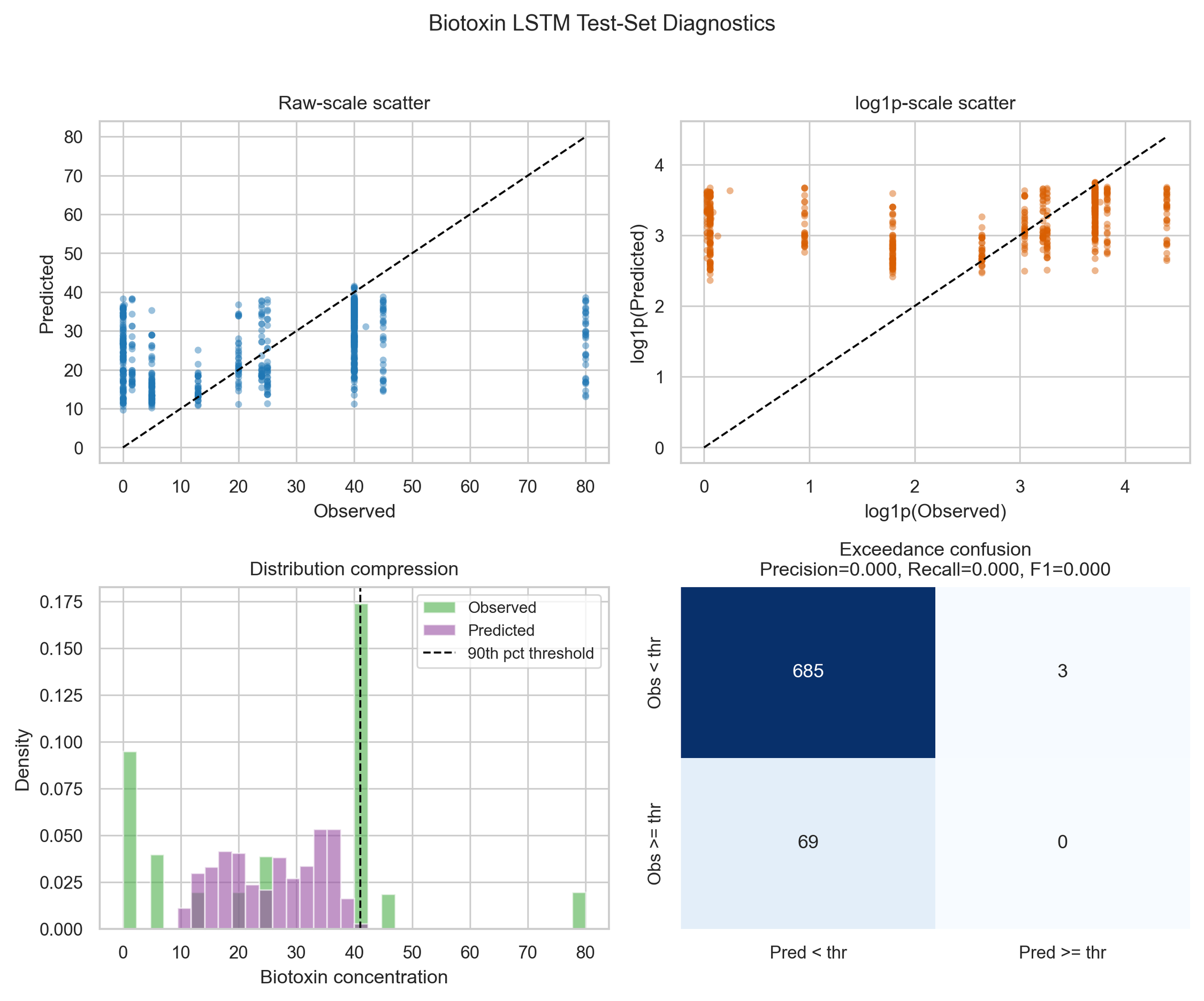

Supplement: S5 Fig — Raw-scale and log1p-scale observed-versus-predicted scatter plots, observed/predicted distribution comparison, and exceedance confusion matrix for the best LSTM model on the held-out biotoxin test set. The diagnostics show that the model captures limited structure in the dominant low-to-moderate concentration regime but fails to recover rare high-end exceedance events. (TIF) [file pone.0351325.s005.tif]

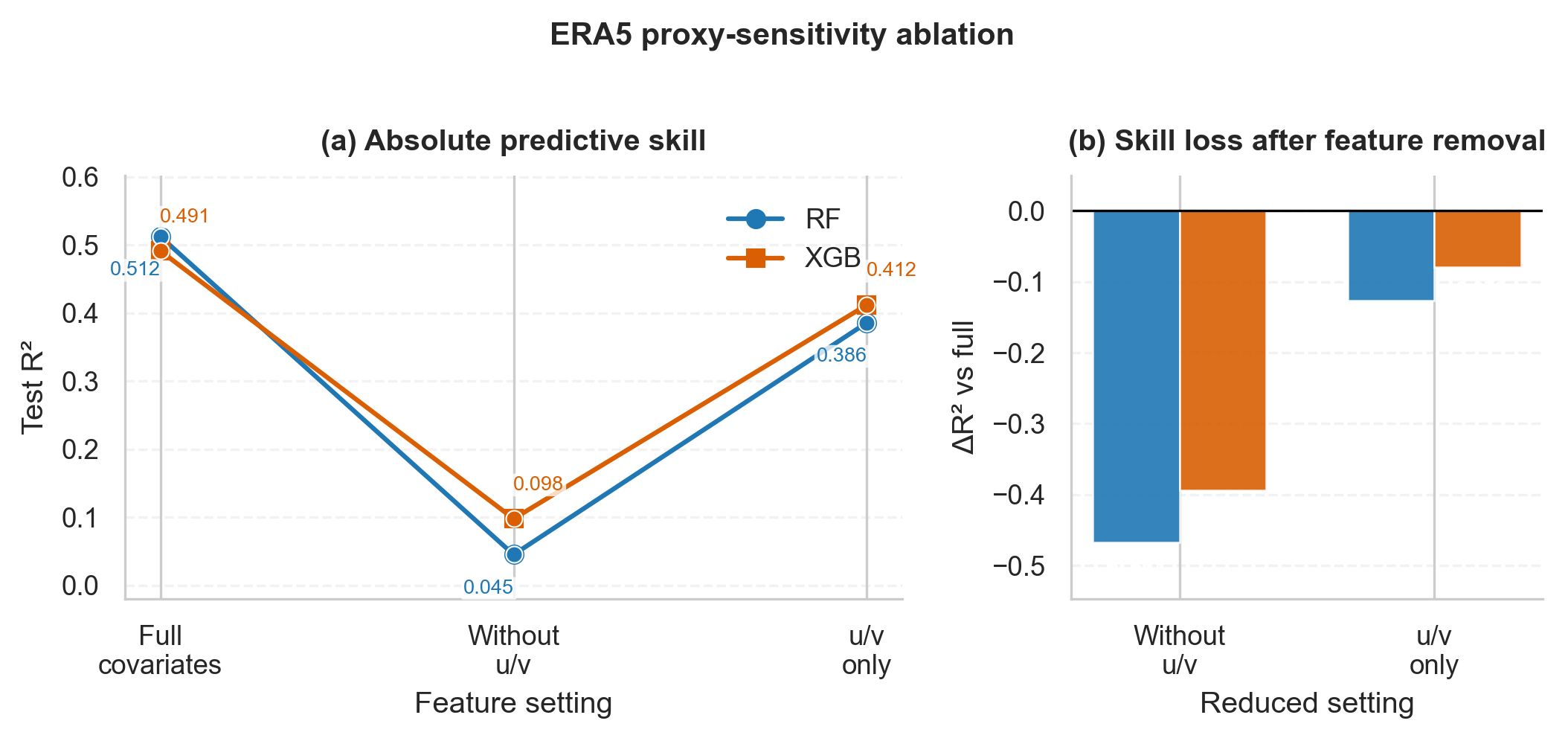

Supplement: S6 Fig — Test-set R² under the full covariate set, without u10/v10, and with u10/v10 only, showing that ERA5 performance depends strongly on immediate wind-component proxies but is not entirely reducible to them. (TIF) [file pone.0351325.s006.tif]
