# Supplementary material for: Cross-dataset benchmarking of machine learning models for marine and atmospheric environmental prediction
Source: PLoS One. 2026 Jun 12;21(6):e0351325. doi: 10.1371/journal.pone.0351325 (PMC13262816; doi:10.1371/journal.pone.0351325)
Supplement: S1 Table — Test-set R² with 95% bootstrap confidence intervals across datasets and models, with dataset-level permutation p-values (XGB; K = 10,000) reported as a sanity check; p-values are computed per dataset and therefore shared across models within the same dataset. (DOCX) [file pone.0351325.s007.docx]

# S1 Table

| Dataset | Model | R² | R² (95% CI) | p-value | MAE | Type |
| --- | --- | --- | --- | --- | --- | --- |
| biotoxin | LASSO | -0.0046 | [-0.018, -0.000] | 0.9953 | 17.7621 | Baseline |
| biotoxin | MEAN | -0.0025 | [-0.014, -0.000] | 0.9953 | 17.7612 | Baseline |
| biotoxin | RIDGE | -0.006 | [-0.021, -0.000] | 0.9953 | 17.7779 | Baseline |
| biotoxin | LSTM | 0.1707 | [0.120, 0.223] | 0.9953 | 14.2095 | Deep Learning |
| biotoxin | TRANSFORMER | -0.0001 | [-0.007, 0.000] | 0.9953 | 17.7713 | Deep Learning |
| biotoxin | RF | -0.0057 | [-0.020, -0.000] | 0.9953 | 17.7741 | Traditional ML |
| biotoxin | SVR | -0.423 | [-0.537, -0.329] | 0.9953 | 17.7231 | Traditional ML |
| biotoxin | XGB | -0.006 | [-0.021, -0.000] | 0.9953 | 17.778 | Traditional ML |
| cast | LASSO | 0.0959 | [0.077, 0.115] | p < 1e-4 | 1301.309 | Baseline |
| cast | MEAN | -0.0 | [-0.002, -0.000] | p < 1e-4 | 1406.9774 | Baseline |
| cast | RIDGE | 0.096 | [0.077, 0.115] | p < 1e-4 | 1301.2664 | Baseline |
| cast | RF | 0.3832 | [0.357, 0.412] | p < 1e-4 | 926.6285 | Traditional ML |
| cast | SVR | 0.0886 | [0.078, 0.098] | p < 1e-4 | 1331.072 | Traditional ML |
| cast | XGB | 0.3805 | [0.354, 0.406] | p < 1e-4 | 959.9495 | Traditional ML |
| cleaned_data | LASSO | -0.0049 | [-0.015, -0.000] | p < 1e-4 | 0.0695 | Baseline |
| cleaned_data | MEAN | -0.0049 | [-0.015, -0.000] | p < 1e-4 | 0.0695 | Baseline |
| cleaned_data | RIDGE | 0.6934 | [0.659, 0.726] | p < 1e-4 | 0.0368 | Baseline |
| cleaned_data | LSTM | 0.3208 | [0.253, 0.377] | p < 1e-4 | 0.0587 | Deep Learning |
| cleaned_data | TRANSFORMER | 0.01 | [-0.005, 0.021] | p < 1e-4 | 0.0689 | Deep Learning |
| cleaned_data | RF | 0.8227 | [0.798, 0.845] | p < 1e-4 | 0.0252 | Traditional ML |
| cleaned_data | SVR | 0.5522 | [0.512, 0.588] | p < 1e-4 | 0.0498 | Traditional ML |
| cleaned_data | XGB | 0.8305 | [0.801, 0.852] | p < 1e-4 | 0.0246 | Traditional ML |
| era5_daily | LASSO | -0.1207 | [-0.135, -0.077] | p < 1e-4 | 1.37 | Baseline |
| era5_daily | MEAN | -0.1741 | [-0.191, -0.149] | p < 1e-4 | 1.4228 | Baseline |
| era5_daily | RIDGE | -0.1328 | [-0.143, -0.075] | p < 1e-4 | 1.3661 | Baseline |
| era5_daily | RF | 0.5125 | [0.489, 0.549] | p < 1e-4 | 0.7749 | Traditional ML |
| era5_daily | SVR | 0.4318 | [0.405, 0.478] | p < 1e-4 | 0.8327 | Traditional ML |
| era5_daily | XGB | 0.4914 | [0.474, 0.536] | p < 1e-4 | 0.8164 | Traditional ML |
| hydrographic | LASSO | -0.1191 | [-0.176, -0.078] | p < 1e-4 | 0.2364 | Baseline |
| hydrographic | MEAN | -0.1191 | [-0.176, -0.078] | p < 1e-4 | 0.2364 | Baseline |
| hydrographic | RIDGE | -0.2702 | [-0.361, -0.198] | p < 1e-4 | 0.2677 | Baseline |
| hydrographic | LSTM | 0.4579 | [0.373, 0.539] | p < 1e-4 | 0.146 | Deep Learning |
| hydrographic | TRANSFORMER | 0.4239 | [0.333, 0.507] | p < 1e-4 | 0.1529 | Deep Learning |
| hydrographic | RF | -0.3536 | [-0.485, -0.248] | p < 1e-4 | 0.2651 | Traditional ML |
| hydrographic | SVR | -0.5874 | [-0.755, -0.435] | p < 1e-4 | 0.2936 | Traditional ML |
| hydrographic | XGB | -0.592 | [-0.740, -0.465] | p < 1e-4 | 0.3021 | Traditional ML |
| processed_seq | LASSO | -0.0 | [-0.004, -0.000] | 1.0000 | 0.0716 | Baseline |
| processed_seq | MEAN | -0.0 | [-0.004, -0.000] | 1.0000 | 0.0716 | Baseline |
| processed_seq | RIDGE | 0.0628 | [0.018, 0.104] | 1.0000 | 0.0673 | Baseline |
| processed_seq | LSTM | 0.5089 | [0.449, 0.561] | 1.0000 | 0.0442 | Deep Learning |
| processed_seq | TRANSFORMER | 0.0048 | [-0.001, 0.007] | 1.0000 | 0.0712 | Deep Learning |
| processed_seq | RF | 0.0404 | [-0.012, 0.086] | 1.0000 | 0.0681 | Traditional ML |
| processed_seq | SVR | -0.0962 | [-0.164, -0.049] | 1.0000 | 0.0788 | Traditional ML |
| processed_seq | XGB | -0.0281 | [-0.088, 0.025] | 1.0000 | 0.07 | Traditional ML |
| rolling_mean | LASSO | -0.0158 | [-0.031, -0.006] | p < 1e-4 | 0.0344 | Baseline |
| rolling_mean | MEAN | -0.0158 | [-0.031, -0.006] | p < 1e-4 | 0.0344 | Baseline |
| rolling_mean | RIDGE | 0.8544 | [0.834, 0.873] | p < 1e-4 | 0.0129 | Baseline |
| rolling_mean | LSTM | 0.6486 | [0.606, 0.688] | p < 1e-4 | 0.0198 | Deep Learning |
| rolling_mean | TRANSFORMER | 0.1455 | [0.127, 0.166] | p < 1e-4 | 0.0322 | Deep Learning |
| rolling_mean | RF | 0.8636 | [0.843, 0.882] | p < 1e-4 | 0.0122 | Traditional ML |
| rolling_mean | SVR | 0.077 | [0.015, 0.131] | p < 1e-4 | 0.0388 | Traditional ML |
| rolling_mean | XGB | 0.8715 | [0.855, 0.886] | p < 1e-4 | 0.012 | Traditional ML |
