# Supplementary material for: Cross-dataset benchmarking of machine learning models for marine and atmospheric environmental prediction
Source: PLoS One. 2026 Jun 12;21(6):e0351325. doi: 10.1371/journal.pone.0351325 (PMC13262816; doi:10.1371/journal.pone.0351325)
Supplement: S2 Table — Dataset-level label-permutation sanity-check p-values using XGBoost with K = 10,000 permutations, reflecting overall dataset predictability rather than model-specific significance. (DOCX) [file pone.0351325.s008.docx]

# S2 Table

| dataset | original_r2 | permuted_r2 | p_value | pass_sanity_check | n_features | n_samples | model_type | n_permutations | split_rule |
| --- | --- | --- | --- | --- | --- | --- | --- | --- | --- |
| biotoxin | 7.258143074895251e-06 | -0.0018970195520711 | 0.9953004699530048 | True | 2 | 4315 | xgb | 10000 | stratified-random-70/15/15 |
| cast | 0.3606442473943826 | -0.0518421472010285 | 9.999000099990002e-05 | True | 12 | 18586 | xgb | 10000 | stratified-random-70/15/15 |
| cleaned_data | 0.6450279681174884 | -0.143264590461494 | 9.999000099990002e-05 | True | 55 | 6646 | xgb | 10000 | chronological-70/15/15 |
| era5_daily | 0.5051622827646264 | -0.0340641272773407 | 9.999000099990002e-05 | True | 8 | 87535 | xgb | 10000 | chronological-70/15/15 |
| hydrographic | -0.7519271365180791 | -0.1813299255718057 | 9.999000099990002e-05 | False | 11 | 3955 | xgb | 10000 | chronological-70/15/15 |
| phyto_long | -1.3513845263072302 | -1.1977139868596012 | 0.3250674932506749 | False | 1 | 70 | xgb | 10000 | stratified-random-70/15/15 |
| phyto_wide | 0.0 | -227.8115066172064 | 1.0 | False | 46 | 374 | xgb | 10000 | stratified-random-70/15/15 |
| processed_seq | -0.0166126944235571 | -0.1244009113222675 | 1.0 | True | 30 | 6833 | xgb | 10000 | chronological-70/15/15 |
| rolling_mean | 0.3781613338580214 | -0.1283366052421322 | 9.999000099990002e-05 | True | 55 | 7527 | xgb | 10000 | chronological-70/15/15 |
