# Supplementary material for: Cross-dataset benchmarking of machine learning models for marine and atmospheric environmental prediction
Source: PLoS One. 2026 Jun 12;21(6):e0351325. doi: 10.1371/journal.pone.0351325 (PMC13262816; doi:10.1371/journal.pone.0351325)
Supplement: S3 Table — Summary of sample size, dimensionality, sample-to-feature ratio, and benchmark-eligibility status for datasets near or below the predefined inclusion threshold, showing why phyto_long and phyto_wide were excluded from the main benchmark and retained for descriptive analysis only. (DOCX) [file pone.0351325.s009.docx]

# S3 Table

| Dataset | Samples | Variables | Sample/Feature Ratio | Benchmark requirement | Meets benchmark requirement | Curse of Dimensionality Risk | Exclusion Reason | Data Quality | Recommendation |
| --- | --- | --- | --- | --- | --- | --- | --- | --- | --- |
| phyto_long | 82 | 1 | 82.0 | N ≥ 500 | False | LOW | Below benchmark inclusion threshold (N < 500) | High quality but insufficient quantity | Descriptive statistics only |
| phyto_wide | 440 | 46 | 9.57 | N ≥ 500 and sample/feature ratio ≥ 10 | False | LOW | Below benchmark inclusion threshold and insufficient sample/feature ratio | High dimensional species data | Descriptive statistics only |
