# Supplementary material for: Cross-dataset benchmarking of machine learning models for marine and atmospheric environmental prediction
Source: PLoS One. 2026 Jun 12;21(6):e0351325. doi: 10.1371/journal.pone.0351325 (PMC13262816; doi:10.1371/journal.pone.0351325)
Supplement: S4 Table — Reported values are mean test-set R² and standard deviation across repeats for each subsample size. (DOCX) [file pone.0351325.s010.docx]

# S4 Table

| dataset | size | R^2^_mean_ | R^2^_std_ | repeats | seed |
| --- | --- | --- | --- | --- | --- |
| era5_daily | 102982 | 0.5447841946275088 | 0.0020272341275821 | 3 | 42 |
| era5_daily | 100000 | 0.5476454744855151 | 0.0010555196352383 | 3 | 42 |
| era5_daily | 50000 | 0.5423079883596131 | 0.0057470634437138 | 3 | 42 |
| era5_daily | 10000 | 0.5244201606368012 | 0.0117133120988686 | 3 | 42 |
| era5_daily | 5000 | 0.4877907122835248 | 0.0407645969261313 | 3 | 42 |
| era5_daily | 1000 | 0.411317616701272 | 0.038261668720199 | 3 | 42 |
