# Supplementary material for: Cross-dataset benchmarking of machine learning models for marine and atmospheric environmental prediction
Source: PLoS One. 2026 Jun 12;21(6):e0351325. doi: 10.1371/journal.pone.0351325 (PMC13262816; doi:10.1371/journal.pone.0351325)
Supplement: S5 Table — RMSE, NRMSE (normalized by the training-set target range), and NSE for all dataset–model combinations; For all datasets, event-based precision/recall/F1 using a fixed threshold defined as the 90th percentile of the training-set target distribution (evaluated on the test set; processed target space). (DOCX) [file pone.0351325.s011.docx]

# S5 Table

| dataset | model | split_rule | train_n | test_n | R^2^ | RMSE | NRMSE | NSE | event_precision | event_recall | event_f1 | event_threshold | Model | Dataset | Split Rule |
| --- | --- | --- | --- | --- | --- | --- | --- | --- | --- | --- | --- | --- | --- | --- | --- |
| cast | rf | stratified-random-70/15/15 | 15305 | 3280 | 0.3832212778909071 | 1201.4294658253182 | 0.1530288454751392 | 0.3832212778909071 | 0.8604651162790697 | 0.1128048780487804 | 0.1994609164420485 | 4125.6 | RF | cast | stratified-random-70/15/15 |
| cast | xgb | stratified-random-70/15/15 | 15305 | 3280 | 0.3804962005758726 | 1204.080643399236 | 0.1533665320849874 | 0.3804962005758726 | 0.573170731707317 | 0.1432926829268292 | 0.2292682926829268 | 4125.6 | XGB | cast | stratified-random-70/15/15 |
| cast | svr | stratified-random-70/15/15 | 15305 | 3280 | 0.0886211256722788 | 1460.4378043458933 | 0.1860193356700921 | 0.0886211256722788 | 0.0 | 0.0 | 0.0 | 4125.6 | SVR | cast | stratified-random-70/15/15 |
| cast | ridge | stratified-random-70/15/15 | 15305 | 3280 | 0.0959558162746766 | 1454.5492003245554 | 0.1852692905775768 | 0.0959558162746766 | 0.0 | 0.0 | 0.0 | 4125.6 | RIDGE | cast | stratified-random-70/15/15 |
| cast | lasso | stratified-random-70/15/15 | 15305 | 3280 | 0.0959495028548215 | 1454.554279259819 | 0.1852699374932899 | 0.0959495028548215 | 0.0 | 0.0 | 0.0 | 4125.6 | LASSO | cast | stratified-random-70/15/15 |
| cast | mean | stratified-random-70/15/15 | 15305 | 3280 | -1.834501404562872e-06 | 1529.7976379852023 | 0.194853857850618 | -1.834501404562872e-06 | 0.0 | 0.0 | 0.0 | 4125.6 | MEAN | cast | stratified-random-70/15/15 |
| biotoxin | rf | chronological-70/15/15 | 3553 | 762 | -0.0057382499927471 | 20.75105319036979 | 0.2361562898642288 | -0.0057382499927471 | 0.0 | 0.0 | 0.0 | 41.0 | RF | biotoxin | chronological-70/15/15 |
| biotoxin | xgb | chronological-70/15/15 | 3553 | 762 | -0.0060448095619134 | 20.754215518759786 | 0.2361922785792623 | -0.0060448095619134 | 0.0 | 0.0 | 0.0 | 41.0 | XGB | biotoxin | chronological-70/15/15 |
| biotoxin | svr | chronological-70/15/15 | 3553 | 762 | -0.4229945070414895 | 24.68307550537814 | 0.2809044668871986 | -0.4229945070414895 | 0.0 | 0.0 | 0.0 | 41.0 | SVR | biotoxin | chronological-70/15/15 |
| biotoxin | ridge | chronological-70/15/15 | 3553 | 762 | -0.0060400780225882 | 20.75416671402394 | 0.2361917231594849 | -0.0060400780225882 | 0.0 | 0.0 | 0.0 | 41.0 | RIDGE | biotoxin | chronological-70/15/15 |
| biotoxin | lasso | chronological-70/15/15 | 3553 | 762 | -0.0045937798167956 | 20.7392430988432 | 0.2360218857271332 | -0.0045937798167956 | 0.0 | 0.0 | 0.0 | 41.0 | LASSO | biotoxin | chronological-70/15/15 |
| biotoxin | mean | chronological-70/15/15 | 3553 | 762 | -0.002513787679804 | 20.71776187160112 | 0.235777419729158 | -0.002513787679804 | 0.0 | 0.0 | 0.0 | 41.0 | MEAN | biotoxin | chronological-70/15/15 |
| biotoxin | lstm | chronological-70/15/15 | 3532 | 757 | 0.1706705917094944 | 18.860930272750647 | 0.2146458435501382 | 0.1706705917094944 | 0.0 | 0.0 | 0.0 | 41.0 | LSTM | biotoxin | chronological-70/15/15 |
| biotoxin | transformer | chronological-70/15/15 | 3532 | 757 | -8.131501089714277e-05 | 20.71177088201689 | 0.2357092395813917 | -8.131501089714277e-05 | 0.0 | 0.0 | 0.0 | 41.0 | TRANSFORMER | biotoxin | chronological-70/15/15 |
| era5_daily | rf | chronological-70/15/15 | 72087 | 15448 | 0.5124633835962307 | 1.1686306660776329 | 0.1237548935804535 | 0.5124633835962307 | 0.827972027972028 | 0.5925925925925926 | 0.690781796966161 | 6.781826591491699 | RF | era5_daily | chronological-70/15/15 |
| era5_daily | xgb | chronological-70/15/15 | 72087 | 15448 | 0.4914402376836627 | 1.19356110190943 | 0.1263949607315525 | 0.4914402376836627 | 0.7916073968705548 | 0.5570570570570571 | 0.6539365452408931 | 6.781826591491699 | XGB | era5_daily | chronological-70/15/15 |
| era5_daily | svr | chronological-70/15/15 | 72087 | 15448 | 0.4318191933149864 | 1.2615862254265953 | 0.1335986412150704 | 0.4318191933149864 | 0.851639344262295 | 0.52002002002002 | 0.6457426973275326 | 6.781826591491699 | SVR | era5_daily | chronological-70/15/15 |
| era5_daily | ridge | chronological-70/15/15 | 72087 | 15448 | -0.1328499698048355 | 1.78139347700877 | 0.1886448529645973 | -0.1328499698048355 | 0.0 | 0.0 | 0.0 | 6.781826591491699 | RIDGE | era5_daily | chronological-70/15/15 |
| era5_daily | lasso | chronological-70/15/15 | 72087 | 15448 | -0.1207317889911159 | 1.7718400101838003 | 0.1876331661206976 | -0.1207317889911159 | 0.0 | 0.0 | 0.0 | 6.781826591491699 | LASSO | era5_daily | chronological-70/15/15 |
| era5_daily | mean | chronological-70/15/15 | 72087 | 15448 | -0.1741003101409275 | 1.8135363312535264 | 0.1920486961307047 | -0.1741003101409275 | 0.0 | 0.0 | 0.0 | 6.781826591491699 | MEAN | era5_daily | chronological-70/15/15 |
| cleaned_data | rf | chronological-70/15/15 | 5473 | 1173 | 0.8226648678149681 | 0.037623937177472 | 0.0935918835260497 | 0.8226648678149681 | 0.8058252427184466 | 0.6240601503759399 | 0.7033898305084746 | 0.224538 | RF | cleaned_data | chronological-70/15/15 |
| cleaned_data | xgb | chronological-70/15/15 | 5473 | 1173 | 0.8305206640783285 | 0.0367811434038404 | 0.0914953816015931 | 0.8305206640783285 | 0.8235294117647058 | 0.7368421052631579 | 0.7777777777777778 | 0.224538 | XGB | cleaned_data | chronological-70/15/15 |
| cleaned_data | svr | chronological-70/15/15 | 5473 | 1173 | 0.5521859181761646 | 0.059788233525571 | 0.1487269490685846 | 0.5521859181761646 | 0.8701298701298701 | 0.5037593984962406 | 0.638095238095238 | 0.224538 | SVR | cleaned_data | chronological-70/15/15 |
| cleaned_data | ridge | chronological-70/15/15 | 5473 | 1173 | 0.6934409365318639 | 0.0494679518507211 | 0.1230546065938337 | 0.6934409365318639 | 0.8095238095238095 | 0.5112781954887218 | 0.6267281105990783 | 0.224538 | RIDGE | cleaned_data | chronological-70/15/15 |
| cleaned_data | lasso | chronological-70/15/15 | 5473 | 1173 | -0.0048502342842839 | 0.0895607080006857 | 0.222787830847477 | -0.0048502342842839 | 0.0 | 0.0 | 0.0 | 0.224538 | LASSO | cleaned_data | chronological-70/15/15 |
| cleaned_data | mean | chronological-70/15/15 | 5473 | 1173 | -0.0048502342842839 | 0.0895607080006857 | 0.222787830847477 | -0.0048502342842839 | 0.0 | 0.0 | 0.0 | 0.224538 | MEAN | cleaned_data | chronological-70/15/15 |
| cleaned_data | lstm | chronological-70/15/15 | 5452 | 1169 | 0.3208241310102541 | 0.0737105462800304 | 0.1833595678607723 | 0.3208241310102541 | 0.6901408450704225 | 0.3684210526315789 | 0.4803921568627451 | 0.22454 | LSTM | cleaned_data | chronological-70/15/15 |
| cleaned_data | transformer | chronological-70/15/15 | 5452 | 1169 | 0.0099879707980331 | 0.0889935931772261 | 0.2213770974557863 | 0.0099879707980331 | 0.0 | 0.0 | 0.0 | 0.22454 | TRANSFORMER | cleaned_data | chronological-70/15/15 |
| rolling_mean | rf | chronological-70/15/15 | 6198 | 1329 | 0.8636350464508002 | 0.0172528258403919 | 0.0673782885095587 | 0.8636350464508002 | 0.8783783783783784 | 0.7428571428571429 | 0.804953560371517 | 0.1674035599999999 | RF | rolling_mean | chronological-70/15/15 |
| rolling_mean | xgb | chronological-70/15/15 | 6198 | 1329 | 0.8714657207672116 | 0.0167501359842508 | 0.0654151096963465 | 0.8714657207672116 | 0.8452380952380952 | 0.8114285714285714 | 0.8279883381924198 | 0.1674035599999999 | XGB | rolling_mean | chronological-70/15/15 |
| rolling_mean | svr | chronological-70/15/15 | 6198 | 1329 | 0.076983026952662 | 0.0448862886017882 | 0.1752965764283116 | 0.076983026952662 | 0.0 | 0.0 | 0.0 | 0.1674035599999999 | SVR | rolling_mean | chronological-70/15/15 |
| rolling_mean | ridge | chronological-70/15/15 | 6198 | 1329 | 0.8543609860159974 | 0.0178298512047289 | 0.0696317733493937 | 0.8543609860159974 | 0.8433734939759037 | 0.8 | 0.8211143695014663 | 0.1674035599999999 | RIDGE | rolling_mean | chronological-70/15/15 |
| rolling_mean | lasso | chronological-70/15/15 | 6198 | 1329 | -0.0158457903454831 | 0.0470893555103231 | 0.183900319324829 | -0.0158457903454831 | 0.0 | 0.0 | 0.0 | 0.1674035599999999 | LASSO | rolling_mean | chronological-70/15/15 |
| rolling_mean | mean | chronological-70/15/15 | 6198 | 1329 | -0.0158457903454831 | 0.0470893555103231 | 0.183900319324829 | -0.0158457903454831 | 0.0 | 0.0 | 0.0 | 0.1674035599999999 | MEAN | rolling_mean | chronological-70/15/15 |
| rolling_mean | lstm | chronological-70/15/15 | 6177 | 1324 | 0.6485794158014138 | 0.0277262894139631 | 0.1082808082986535 | 0.6485794158014138 | 0.7368421052631579 | 0.56 | 0.6363636363636364 | 0.1685714285714285 | LSTM | rolling_mean | chronological-70/15/15 |
| rolling_mean | transformer | chronological-70/15/15 | 6177 | 1324 | 0.1455481657542898 | 0.0432336544630776 | 0.1688424650364201 | 0.1455481657542898 | 0.0 | 0.0 | 0.0 | 0.1685714285714285 | TRANSFORMER | rolling_mean | chronological-70/15/15 |
| processed_seq | rf | chronological-70/15/15 | 5627 | 1206 | 0.0403983903806772 | 0.0881864875590094 | 0.2199164278279536 | 0.0403983903806772 | 0.0 | 0.0 | 0.0 | 0.25 | RF | processed_seq | chronological-70/15/15 |
| processed_seq | xgb | chronological-70/15/15 | 5627 | 1206 | -0.0281245160105227 | 0.0912807960445326 | 0.2276329078417271 | -0.0281245160105227 | 0.75 | 0.0545454545454545 | 0.1016949152542373 | 0.25 | XGB | processed_seq | chronological-70/15/15 |
| processed_seq | svr | chronological-70/15/15 | 5627 | 1206 | -0.096242001410119 | 0.0942561681258334 | 0.2350527883437243 | -0.096242001410119 | 0.0 | 0.0 | 0.0 | 0.25 | SVR | processed_seq | chronological-70/15/15 |
| processed_seq | ridge | chronological-70/15/15 | 5627 | 1206 | 0.0628256218237706 | 0.0871498742112163 | 0.2173313571352028 | 0.0628256218237706 | 0.0 | 0.0 | 0.0 | 0.25 | RIDGE | processed_seq | chronological-70/15/15 |
| processed_seq | lasso | chronological-70/15/15 | 5627 | 1206 | -1.630933778162813e-05 | 0.0900243724722005 | 0.2244996819755624 | -1.630933778162813e-05 | 0.0 | 0.0 | 0.0 | 0.25 | LASSO | processed_seq | chronological-70/15/15 |
| processed_seq | mean | chronological-70/15/15 | 5627 | 1206 | -1.630933778162813e-05 | 0.0900243724722005 | 0.2244996819755624 | -1.630933778162813e-05 | 0.0 | 0.0 | 0.0 | 0.25 | MEAN | processed_seq | chronological-70/15/15 |
| processed_seq | lstm | chronological-70/15/15 | 5606 | 1202 | 0.508891357782137 | 0.0631434261969523 | 0.157464903234295 | 0.508891357782137 | 0.8571428571428571 | 0.4363636363636363 | 0.5783132530120482 | 0.25 | LSTM | processed_seq | chronological-70/15/15 |
| processed_seq | transformer | chronological-70/15/15 | 5606 | 1202 | 0.0047838915120758 | 0.0898872416149327 | 0.2241577097629246 | 0.0047838915120758 | 0.0 | 0.0 | 0.0 | 0.25 | TRANSFORMER | processed_seq | chronological-70/15/15 |
| hydrographic | rf | chronological-70/15/15 | 3257 | 698 | -0.3535892024707496 | 0.3505751974319365 | 0.2434549982166225 | -0.3535892024707496 | 0.0714285714285714 | 0.0238095238095238 | 0.0357142857142857 | 1.04 | RF | hydrographic | chronological-70/15/15 |
| hydrographic | xgb | chronological-70/15/15 | 3257 | 698 | -0.5920333238047253 | 0.3802014979434049 | 0.2640288180162534 | -0.5920333238047253 | 0.0652173913043478 | 0.0357142857142857 | 0.0461538461538461 | 1.04 | XGB | hydrographic | chronological-70/15/15 |
| hydrographic | svr | chronological-70/15/15 | 3257 | 698 | -0.5874246373603613 | 0.3796507872862881 | 0.2636463800599223 | -0.5874246373603613 | 0.0612244897959183 | 0.0357142857142857 | 0.0451127819548872 | 1.04 | SVR | hydrographic | chronological-70/15/15 |
| hydrographic | ridge | chronological-70/15/15 | 3257 | 698 | -0.2701621495515165 | 0.3395997250727927 | 0.2358331424116616 | -0.2701621495515165 | 0.0 | 0.0 | 0.0 | 1.04 | RIDGE | hydrographic | chronological-70/15/15 |
| hydrographic | lasso | chronological-70/15/15 | 3257 | 698 | -0.1190700512801266 | 0.3187618826991438 | 0.2213624185410721 | -0.1190700512801266 | 0.0 | 0.0 | 0.0 | 1.04 | LASSO | hydrographic | chronological-70/15/15 |
| hydrographic | mean | chronological-70/15/15 | 3257 | 698 | -0.1190700512801266 | 0.3187618826991438 | 0.2213624185410721 | -0.1190700512801266 | 0.0 | 0.0 | 0.0 | 1.04 | MEAN | hydrographic | chronological-70/15/15 |
| hydrographic | lstm | chronological-70/15/15 | 3236 | 694 | 0.457899759252326 | 0.2221837551169694 | 0.1542942743867843 | 0.457899759252326 | 0.8 | 0.1904761904761904 | 0.3076923076923077 | 1.04 | LSTM | hydrographic | chronological-70/15/15 |
| hydrographic | transformer | chronological-70/15/15 | 3236 | 694 | 0.4239200464411409 | 0.2290413442252347 | 0.1590564890453019 | 0.4239200464411409 | 0.6153846153846154 | 0.0952380952380952 | 0.1649484536082474 | 1.04 | TRANSFORMER | hydrographic | chronological-70/15/15 |
