# Supplementary material for: Cross-dataset benchmarking of machine learning models for marine and atmospheric environmental prediction
Source: PLoS One. 2026 Jun 12;21(6):e0351325. doi: 10.1371/journal.pone.0351325 (PMC13262816; doi:10.1371/journal.pone.0351325)
Supplement: S6 Table — Cutoff dates and train/validation/test ratios for all time-series datasets. (DOCX) [file pone.0351325.s012.docx]

# S6 Table

| Dataset | Split Rule | Train Range | Val Range | Test Range | Period |
| --- | --- | --- | --- | --- | --- |
| cleaned_data | time-ordered | 1992-08-13 to 2018-05-24 | 2018-05-24 to 2019-08-16 | 2019-08-16 to 2021-10-04 | 1992-08-13 to 2021-10-04 |
| rolling_mean | time-ordered | 1992-08-16 to 2018-04-10 | 2018-04-10 to 2019-07-24 | 2019-07-24 to 2021-10-04 | 1992-08-16 to 2021-10-04 |
| processed_seq | time-ordered | 1970-02-28 to 1992-03-13 | 1992-03-14 to 1998-12-06 | 1998-12-07 to 2005-03-07 | 1970-02-28 to 2005-03-07 |
| cast | random | nan | nan | nan | nan |
| era5_daily | time-ordered | 2024-01-02 to 2025-01-09 | 2025-01-09 to 2025-03-12 | 2025-03-12 to 2025-06-15 | 2024-01-02 to 2025-06-15 |
| biotoxin | time-ordered | 2013-12-03 to 2022-04-05 | 2022-04-05 to 2023-03-13 | 2023-03-13 to 2023-06-22 | 2013-12-03 to 2023-06-22 |
| hydrographic | time-ordered | 2014-05-20 to 2021-01-12 | 2021-01-12 to 2022-06-06 | 2022-06-06 to 2023-12-18 | 2014-05-20 to 2023-12-18 |
| phyto_long | time-ordered | 2014-05-27 to 2021-05-03 | 2021-06-01 to 2021-12-16 | 2022-01-18 to 2022-12-13 | 2014-05-27 to 2022-12-13 |
| phyto_wide | random | nan | nan | nan | nan |
