# Supplementary material for: Cross-dataset benchmarking of machine learning models for marine and atmospheric environmental prediction
Source: PLoS One. 2026 Jun 12;21(6):e0351325. doi: 10.1371/journal.pone.0351325 (PMC13262816; doi:10.1371/journal.pone.0351325)
Supplement: S7 Table — Region descriptions and coordinate bounds for each dataset. For datasets without geolocation metadata (e.g., site-aggregated or non-georeferenced products), coordinate fields are reported as NA in S7 Table. (DOCX) [file pone.0351325.s013.docx]

# S7 Table

| Dataset | Latitude Column | Longitude Column | Lat Min | Lat Max | Lon Min | Lon Max |
| --- | --- | --- | --- | --- | --- | --- |
| biotoxin | LATITUDE (degrees North) | LONGITUDE (degrees East) | 43.343565 | 43.355712 | -2.448422 | -2.444065 |
| cast | Lat_Dec | Lon_Dec | 23.65 | 39.916666 | -128.966666 | -110.591666 |
| cleaned_data | G2latitude | G2longitude | -35.508 | 50.0163 | 120.79 | 179.13 |
| era5_daily | latitude | longitude | 37.0 | 41.0 | 117.0 | 122.0 |
| hydrographic | LATITUDE (degrees North) | LONGITUDE (degrees East) | 43.35685 | 43.35685 | -2.448633 | -2.448633 |
| phyto_long | nan | nan | nan | nan | nan | nan |
| phyto_wide | LATITUDE (degrees North) | LONGITUDE (degrees East) | 43.35685 | 43.35685 | -2.448633 | -2.448633 |
| processed_seq | nan | nan | nan | nan | nan | nan |
| rolling_mean | G2latitude | G2longitude | -42.86917142857143 | 47.44744285714285 | 95.54142857142855 | 178.9567 |
