# Supplementary material for: Cross-dataset benchmarking of machine learning models for marine and atmospheric environmental prediction
Source: PLoS One. 2026 Jun 12;21(6):e0351325. doi: 10.1371/journal.pone.0351325 (PMC13262816; doi:10.1371/journal.pone.0351325)
Supplement: S8 Table — Performance of a model trained on one chlorophyll-a dataset and evaluated directly on another (cleaned_data ↔ rolling_mean), reporting R²/MAE/RMSE and illustrating the impact of domain shift on predictive accuracy. (DOCX) [file pone.0351325.s014.docx]

# S8 Table

| train_dataset | test_dataset | n_features | R^2^ | MAE | RMSE |
| --- | --- | --- | --- | --- | --- |
| cleaned_data | rolling_mean | 69 | 0.728668633308857 | 0.0194088145005428 | 0.0240550803811049 |
| rolling_mean | cleaned_data | 69 | 0.6455011228537056 | 0.0371239272174399 | 0.051292917041945 |
