# Supplementary material for: Cross-dataset benchmarking of machine learning models for marine and atmospheric environmental prediction
Source: PLoS One. 2026 Jun 12;21(6):e0351325. doi: 10.1371/journal.pone.0351325 (PMC13262816; doi:10.1371/journal.pone.0351325)
Supplement: S9 Table — Test-set performance of RF, XGB, and LSTM under chronological versus random 70/15/15 splits, showing that random splitting yields systematically more optimistic scores. (DOCX) [file pone.0351325.s015.docx]

# S9 Table

| dataset | model | split_mode | split_rule | n_train | n_val | n_test | R² | MAE | RMSE |
| --- | --- | --- | --- | --- | --- | --- | --- | --- | --- |
| hydrographic | RF | chronological | chronological-70/15/15 | 3257 | 698 | 698 | -0.3535892024707496 | 0.2651143266475644 | 0.3505751974319365 |
| hydrographic | XGB | chronological | chronological-70/15/15 | 3257 | 698 | 698 | -0.5920333238047253 | 0.3021242434447124 | 0.3802014979434049 |
| hydrographic | LSTM | chronological | chronological-70/15/15 | 3236 | 693 | 694 | 0.457899759252326 | 0.1460028475325457 | 0.2221837551169694 |
| hydrographic | RF | random | stratified-random-70/15/15 | 3257 | 697 | 699 | 0.5477289876111017 | 0.1695415090829712 | 0.2259597623913458 |
| hydrographic | XGB | random | stratified-random-70/15/15 | 3257 | 697 | 699 | 0.8609790628340386 | 0.0820280014833326 | 0.125277142466007 |
| hydrographic | LSTM | random | sequence-stratified-random-70/15/15 | 3236 | 693 | 694 | 0.6728265030275722 | 0.1265017664682667 | 0.1908500844556494 |
