# Supplementary material for: Cross-dataset benchmarking of machine learning models for marine and atmospheric environmental prediction
Source: PLoS One. 2026 Jun 12;21(6):e0351325. doi: 10.1371/journal.pone.0351325 (PMC13262816; doi:10.1371/journal.pone.0351325)
Supplement: S10 Table — Test-set sample size, threshold, R², MAE, RMSE, and exceedance precision/recall/F1 for MEAN, RF, XGB, and LSTM on the biotoxin dataset. For the LSTM, the test-set count is slightly smaller because sequence construction removes a small number of boundary samples near the split edges. (DOCX) [file pone.0351325.s016.docx]

# S10 Table

| dataset | model | n_test | threshold | R² | MAE | RMSE | event_precision | event_recall | event_f1 | tp | tn | fp | fn |
| --- | --- | --- | --- | --- | --- | --- | --- | --- | --- | --- | --- | --- | --- |
| biotoxin | MEAN | 762 | 41.0 | -0.002513787679804 | 17.761180990076774 | 20.71776187160112 | 0.0 | 0.0 | 0.0 | 0 | 691 | 0 | 71 |
| biotoxin | RF | 762 | 41.0 | -0.0057382499927471 | 17.774050103933526 | 20.75105319036979 | 0.0 | 0.0 | 0.0 | 0 | 691 | 0 | 71 |
| biotoxin | XGB | 762 | 41.0 | -0.0060448095619134 | 17.77795475997324 | 20.754215518759786 | 0.0 | 0.0 | 0.0 | 0 | 691 | 0 | 71 |
| biotoxin | LSTM | 757 | 41.0 | 0.1706705917094944 | 14.209455996655723 | 18.860930272750647 | 0.0 | 0.0 | 0.0 | 0 | 685 | 3 | 69 |
