# Supplementary material for: Cross-dataset benchmarking of machine learning models for marine and atmospheric environmental prediction
Source: PLoS One. 2026 Jun 12;21(6):e0351325. doi: 10.1371/journal.pone.0351325 (PMC13262816; doi:10.1371/journal.pone.0351325)
Supplement: S11 Table — Test-set R², MAE, RMSE, and ΔR² relative to the full-feature setting for RF and XGB under three ERA5 predictor settings: full covariates, without u10/v10, and u10/v10 only. (DOCX) [file pone.0351325.s017.docx]

# S11 Table

| dataset | model | setting | n_features | features | R² | MAE | RMSE | delta_r2_vs_full |
| --- | --- | --- | --- | --- | --- | --- | --- | --- |
| era5_daily | RF | full | 8 | latitude,longitude,number,u10,v10,msl,tcc,ssr_flux | 0.5124633835962307 | 0.7748843695916687 | 1.1686306660776329 | 0.0 |
| era5_daily | RF | no_uv | 6 | latitude,longitude,number,msl,tcc,ssr_flux | 0.0452587393466595 | 1.2600266047692308 | 1.6353718009367748 | -0.4672046442495712 |
| era5_daily | RF | uv_only | 2 | u10,v10 | 0.3860390176460898 | 0.86352131474157 | 1.311426771601539 | -0.1264243659501409 |
| era5_daily | XGB | full | 8 | latitude,longitude,number,u10,v10,msl,tcc,ssr_flux | 0.4914402376836627 | 0.8163771781470416 | 1.19356110190943 | 0.0 |
| era5_daily | XGB | no_uv | 6 | latitude,longitude,number,msl,tcc,ssr_flux | 0.0979746797941735 | 1.2132195195328812 | 1.5895823183291873 | -0.3934655578894891 |
| era5_daily | XGB | uv_only | 2 | u10,v10 | 0.412180653099605 | 0.8608043037397388 | 1.283203681579843 | -0.0792595845840576 |
