# Supplementary material for: Cross-dataset benchmarking of machine learning models for marine and atmospheric environmental prediction
Source: PLoS One. 2026 Jun 12;21(6):e0351325. doi: 10.1371/journal.pone.0351325 (PMC13262816; doi:10.1371/journal.pone.0351325)
Supplement: S12 Table — Test-set performance of persistence and ARIMA on rolling_mean, processed_seq, and era5_daily, together with the corresponding best benchmark model for reference. For era5_daily, the comparison is contextual because ARIMA is fit to a univariate panel-by-date series rather than to the full multivariate covariate set. (DOCX) [file pone.0351325.s018.docx]

# S12 Table

| dataset | baseline_method | split_rule | series_type | n_train | n_val | n_test | R² | MAE | RMSE | arima_order | benchmark_best_model | benchmark_best_r2 |
| --- | --- | --- | --- | --- | --- | --- | --- | --- | --- | --- | --- | --- |
| rolling_mean | PERSISTENCE | chronological-70/15/15 | univariate | 6198 | 1328 | 1329 | 0.3965768634049059 | 0.0239578630549285 | 0.0362927463031174 | nan | XGB | 0.8714657207672116 |
| rolling_mean | ARIMA | chronological-70/15/15 | univariate | 6198 | 1328 | 1329 | -0.0086949002821323 | 0.0345422017231284 | 0.0469233236707818 | (2, 0, 1) | XGB | 0.8714657207672116 |
| processed_seq | PERSISTENCE | chronological-70/15/15 | univariate | 5627 | 1206 | 1206 | -0.2773012279868951 | 0.0701160862354892 | 0.1017426923963243 | nan | LSTM | 0.508891357782137 |
| processed_seq | ARIMA | chronological-70/15/15 | univariate | 5627 | 1206 | 1206 | -0.0001700750188424 | 0.0712371097765164 | 0.090031293422756 | (2, 0, 1) | LSTM | 0.508891357782137 |
| era5_daily | PERSISTENCE | panel-by-date-70/15/15 | location-wise panel | 358 | 77 | 77 | 0.0081672771004932 | 1.295141381075273 | 1.6720783273616755 | nan | RF | 0.5124633835962307 |
| era5_daily | ARIMA | panel-by-date-70/15/15 | location-wise panel | 358 | 77 | 77 | 0.3454600688243171 | 1.0495893694495235 | 1.3583305696911607 | (1, 1, 1) | RF | 0.5124633835962307 |
